# Prospective Assessment of SARS-CoV-2 Seroconversion (PASS) study: an observational cohort study of SARS-CoV-2 infection and vaccination in healthcare workers

**DOI:** 10.1186/s12879-021-06233-1

**Published:** 2021-06-09

**Authors:** Belinda M. Jackson-Thompson, Emilie Goguet, Eric D. Laing, Cara H. Olsen, Simon Pollett, K. Monique Hollis-Perry, Santina E. Maiolatesi, Luca Illinik, Kathleen F. Ramsey, Anatalio E. Reyes, Yolanda Alcorta, Mimi A. Wong, Julian Davies, Orlando Ortega, Edward Parmelee, Alyssa R. Lindrose, Matthew Moser, Elizabeth Graydon, Andrew G. Letizia, Christopher A. Duplessis, Anuradha Ganesan, Kathleen P. Pratt, Allison M. Malloy, David W. Scott, Stephen K. Anderson, Andrew L. Snow, Clifton L. Dalgard, John H. Powers, David Tribble, Timothy H. Burgess, Christopher C. Broder, Edward Mitre

**Affiliations:** 1 Department of Microbiology and Immunology, Uniformed Services University of the Health Science, Bethesda, MD USA; 2 Henry M. Jackson Foundation for the Advancement of Military Medicine, Inc, Bethesda, MD USA; 3 Department of Preventive Medicine & Biostatistics, Uniformed Services University of the Health Sciences, Bethesda, USA; 4 Infectious Disease Clinical Research Program, Department of Preventive Medicine and Biostatistics, Uniformed Services University of the Health Sciences, Bethesda, MD USA; 5 Clinical Trials Center, Naval Medical Research Center, Silver Spring, MD USA; 6 General Dynamics Information Technology, Falls Church, VA USA; 7 Infectious Disease Directorate, Naval Medical Research Center, Silver Spring, MD USA; 8 Department of Medicine, Uniformed Services University of the Health Sciences, Bethesda, MD USA; 9 Department of Pediatrics, Uniformed Services University of the Health Sciences, Bethesda, MD USA; 10 Basic Science Program, Frederick National Laboratory for Cancer Research, Frederick, MD USA; 11 Department of Pharmacology and Molecular Therapeutics, Uniformed Services University of the Health Sciences, Bethesda, MD USA; 12 Department of Anatomy, Physiology, and Genetics, and The American Genome Center, Uniformed Services University of the Health Sciences, Bethesda, MD USA; 13 Clinical Research Directorate, Leidos Biomedical Research, Inc, Frederick National Laboratory for Cancer Research, Frederick, MD USA

**Keywords:** SARS-CoV-2, COVID-19, Coronavirus, Immune-response, Cross-reactivity, Prospective study

## Abstract

**Background:**

SARS-CoV-2 is a recently emerged pandemic coronavirus (CoV) capable of causing severe respiratory illness. However, a significant number of infected people present as asymptomatic or pauci-symptomatic. In this prospective assessment of at-risk healthcare workers (HCWs) we seek to determine whether pre-existing antibody or T cell responses to previous seasonal human coronavirus (HCoV) infections affect immunological or clinical responses to SARS-CoV-2 infection or vaccination.

**Methods:**

A cohort of 300 healthcare workers, confirmed negative for SARS-CoV-2 exposure upon study entry, will be followed for up to 1 year with monthly serology analysis of IgM and IgG antibodies against the spike proteins of SARS-CoV-2 and the four major seasonal human coronavirus - HCoV-OC43, HCoV-HKU1, HCoV-229E, and HCoV-NL63. Participants will complete monthly questionnaires that ask about Coronavirus Disease 2019 (COVID-19) exposure risks, and a standardized, validated symptom questionnaire (scoring viral respiratory disease symptoms, intensity and severity) at least twice monthly and any day when any symptoms manifest. SARS-CoV-2 PCR testing will be performed any time participants develop symptoms consistent with COVID-19. For those individuals that seroconvert and/or test positive by SARS-CoV-2 PCR, or receive the SARS-CoV-2 vaccine, additional studies of T cell activation and cytokine production in response to SARS-CoV-2 peptide pools and analysis of Natural Killer cell numbers and function will be conducted on that participant’s cryopreserved baseline peripheral blood mononuclear cells (PBMCs). Following the first year of this study we will further analyze those participants having tested positive for COVID-19, and/or having received an authorized/licensed SARS-CoV-2 vaccine, quarterly (year 2) and semi-annually (years 3 and 4) to investigate immune response longevity.

**Discussion:**

This study will determine the frequency of asymptomatic and pauci-symptomatic SARS-CoV-2 infection in a cohort of at-risk healthcare workers. Baseline and longitudinal assays will determine the frequency and magnitude of anti-spike glycoprotein antibodies to the seasonal HCoV-OC43, HCoV-HKU1, HCoV-229E, and HCoV-NL63, and may inform whether pre-existing antibodies to these human coronaviruses are associated with altered COVID-19 disease course. Finally, this study will evaluate whether pre-existing immune responses to seasonal HCoVs affect the magnitude and duration of antibody and T cell responses to SARS-CoV-2 vaccination, adjusting for demographic covariates.

**Supplementary Information:**

The online version contains supplementary material available at 10.1186/s12879-021-06233-1.

## Background

SARS-CoV-2 is a recently emerged pandemic coronavirus (CoV) capable of causing severe respiratory illness (COVID-19), with over 100 million people infected worldwide and over 2 million deaths as of February 9, 2021 [1]. The clinical manifestations of COVID-19 range widely in severity, from minimal to life-threatening respiratory failure and multisystem sequelae [2]. Some of the key unknowns of SARS-CoV-2 infection include the frequency of symptomatic versus asymptomatic/pauci-symptomatic infection, and whether pre-existing antibody or T cell responses to the seasonal HCoVs affect immunological or clinical responses to SARS-CoV-2 infection. The goal of the Prospective Assessment of SARS-CoV-2 Seroconversion (PASS) study is to better understand the symptomatic frequency, clinical manifestations, and immunologic responses to COVID-19, as well as what immunological factors might affect SARS-CoV-2 vaccine responses. The study will focus on a cohort of healthcare workers (HCWs) at the Walter Reed National Military Medical Center (WRNMMC).

### Study aims

#### Frequency of asymptomatic and pauci-symptomatic infections

The exact frequency with which SARS-CoV-2 causes asymptomatic and pauci-symptomatic infections remains unclear, though increasing evidence suggests that mild disease is common. A point prevalence study demonstrated that 56% of residents in a nursing facility who tested PCR positive for SARS-CoV-2 were asymptomatic at time of testing [3]. A mathematical modeling study based on various assumptions estimated that actual numbers of infected individuals in China were likely 10-fold greater than the number of confirmed cases [4], and a serological survey conducted in California suggested that under-ascertainment rates in that region may be as high as 50- to 80-fold [5]. Understanding rates of asymptomatic infections in HCW populations is important because HCWs are at increased risk for SARS-CoV-2 infection [3, 6] and, when infected, pose a transmission risk to other HCWs as well as uninfected patients. Thus, the first aim of this study is to determine the frequency of asymptomatic and pauci-symptomatic SARS-CoV-2 infection in a cohort of HCWs.

A major limitation of cross-sectional studies for SARS-CoV-2 antibodies is recall bias, in that individuals may not recall mild symptoms they had during the time of infection. By conducting an observational cohort study in a prospective fashion in which study participants fill out an online standardized symptom questionnaire every day they have symptoms different than their usual baseline health, and by using monthly serology surveillance to define the presence of host response to infection, we should be able to obtain an accurate assessment of the frequency of true symptomatic infection as well as identify the most common symptoms present in mild disease. These associative findings will improve the assumptions used in epidemiologic modeling and will inform strategies to rapidly identify, test, and quarantine individuals with possible SARS-CoV-2 infection.

#### Potential effects of pre-existing coronavirus antibodies on SARS-CoV-2 disease severity

Another key unknown is whether pre-existing antibodies to seasonal HCoV affect SARS-CoV-2 infection pathogenesis. While it is clear that certain co-morbidities increase risk for severe COVID-19 disease, we do not yet understand why some individuals with few or no co-morbidities become very ill. One possibility is an aberrant immune response caused by pre-existing cross-reactive CoV antibodies exacerbates disease.

SARS-CoV-2 is a member of the genus *Betacoronavirus* which includes seasonal human coronaviruses HCoV-OC43 and HCoV-HKU1, both causative agents of the common-cold. Thus, infection with human coronaviruses is common [7–10] and a recent longitudinal study of 10 individuals demonstrated that antibody levels against human coronaviruses fluctuate over time, likely due to recurrent exposures [11]. The SARS-CoV-2 spike glycoprotein is antigenically-related to the spike proteins of HCoV-OC43 and HCoV-HKU1, sharing 30–40% identity and similarity. A higher percentage of conservation is observed in the S2 subunit region that contains heptad repeats and mediates cell fusion, compared to the more variable S1 subunit region containing the receptor-binding domain (RBD) [12, 13]. Cross-reactive antibodies to native-like SARS-CoV-2 S glycoprotein have been identified in 5–10% of sera collected prior to the emergence of SARS-CoV-2 [13–15]. In fact, the pre-existing B cells from uninfected individuals displayed reactivity with SARS-CoV-2 S glycoprotein S2 subunit and SARS-CoV-2 infected individuals developed antibodies that were cross-reactive with HCoV S glycoprotein epitopes [13]. However, the effect any pre-existing HCoV antibodies may have on disease outcomes remains unknown.

If pre-existing cross-reactive antibodies are able to bind to the SARS-CoV-2 S glycoprotein without neutralizing it, this may facilitate viral entry into immune cells, a phenomenon known as antibody-dependent enhancement (ADE). ADE is well characterized in dengue virus infection and represents a key concern in SARS-CoV-2 infection [16, 17]. This phenomenon has been implicated by some investigators as a contributing factor in severe cases of SARS-CoV infection [18]. The role of pre-existing HCoV-induced antibodies in COVID-19 clinical status or ADE has not been directly examined and remains unknown [19]. Another potentially deleterious effect that pre-existing cross-reactive antibodies may have during SARS-CoV-2 infection is induction of an inflammatory response that does not effectively control the infection [20]. Known as immune enhancement, this phenomenon has been observed with respiratory syncytial virus, and may be driven by non-neutralizing titers of cross-reactive antibodies as well as aberrant memory T cell responses that induce a T-helper 2 response [20]. As such, in addition to obtaining baseline measures of pre-existing cross-reactive CoV antibodies, we will also evaluate whether baseline cross-reactive T cell responses to major SARS-CoV-2 antigens are associated with altered disease severity.

Alternatively, it is quite possible that pre-existing antibodies against other coronaviruses have a beneficial effect on the outcome of SARS-CoV-2 infection. For instance, if conserved S glycoprotein S2 subunit epitopes common between SARS-CoV-2 and other HCoVs are essential for viral function, pre-existing antibodies may have a cross-protective effect. Indeed, some scientists speculate that children are relatively protected from severe COVID-19 due to potential pre-existing high titers to common coronaviruses [21]. Additionally, a recent study suggests that individuals with documented seasonal HCoV infections in the past 5 years exhibit decreased rates of ICU admission and mortality when infected with SARS-CoV-2 compared to those without a history of recent seasonal HCoV infection [22]. Understanding whether pre-existing cross-reactive CoV antibodies are associated with better or worse disease outcomes will have important implications both for vaccine design as well as antibody therapy. The second aim of this study is to investigate the serological footprint of HCoV-OC43, HCoV-HKU1, HCoV-229E, and HCoV-NL63 through active sero-surveillance, and determine whether pre-existing antibodies to these HCoVs are associated with altered COVID-19 disease course**.**

By conducting this study prospectively, we will be able to determine baseline levels of antibodies to the S glycoproteins of the common HCoVs and evaluate changes in the titers over time. As study participants become infected, we will then be able to evaluate whether high titers of antibodies against the seasonal coronaviruses (as well as presence of baseline coronavirus-specific T cell responses) are associated with increased or decreased severity of COVID-19 disease. Of note, in addition to evaluating baseline adaptive immune responses, we will also investigate baseline natural killer (NK) cell frequency and function given the critical importance of NK cells in the early control of viral infections [23, 24].

#### Potential effects of pre-existing immune responses to seasonal coronaviruses on SARS-CoV-2 antibody and T cell responses

It is increasingly recognized that prior exposure to one virus can affect immune responses to vaccination or infection with an antigenically-related virus [25]. For example, it has been shown that vaccination with one strain of influenza vaccine results in reduced antibody responses to vaccination with a different strain [26]. Known as “original antigenic sin,” this hypothesis posits that antibody and T cell responses are imprinted against the first antigen to which a person is exposed, resulting in a reduced ability to develop robust immune responses when exposed to a slightly different version of the same antigen [27]. In addition to the hypothesis of original antigenic sin, factors such as antigenic seniority [28] and antibody landscapes [29] can also affect the development of immune responses to vaccination. For example, Kucharski et al. found that the temporal order in which individuals are exposed to influenza strains drives the duration of antibody response, with the response against antigens encountered earlier in life being more durable [28].

Most people have pre-existing immunity to seasonal HCoVs. However, it is unknown whether immune imprinting may have occurred and/or have had an effect on the development of humoral immunity to SARS-CoV-2. Using multiplex antibody testing we will prospectively determine baseline seasonal HCoV antibody levels and through monthly serology surveillance be able to identify any induction or “back-boosting” of HCoV memory following SARS-CoV-2 infection/seroconversion and/or vaccination. As such, we will be able to correlate if HCoV imprinting has any effect on the development of neutralizing antibodies or the longevity of protective antibodies developed by natural infection or vaccination. Thus, the third aim of this study is to determine if pre-existing immune responses to seasonal HCoVs affect magnitude and duration of antibody and T cell responses to SARS-CoV-2 vaccination.

Studying antibody and T cell reactivity after COVID-19 vaccination will provide information on whether pre-existing immune responses to seasonal HCoVs affect COVID-19 vaccine efficacy. If study participants receive different COVID-19 vaccines, we may be able to evaluate if there are differences in antibody or T cell responses amongst the different vaccines. Investigations conducted on pre-vaccination samples may also inform whether there are specific factors of baseline immunity (innate or adaptive) that are associated with robust antibody responses to SARS-CoV-2 vaccination. This aim will also permit a descriptive characterization of the average durability and magnitude of vaccine induced SARS-CoV-2 immunity in HCWs. Such descriptive analyses will contribute knowledge to outstanding SARS-CoV-2 vaccinology questions regarding frequency of vaccine boosting in this high risk population. Depending on the intensity of circulating SARS-CoV-2 in the U.S. population over the next few years, this long-term study may also be able to inform whether there is a risk of immunopathology from sub-neutralizing titers.

## Methods/design

In this study we seek to address the following central aims: (1) determine the frequency of asymptomatic and pauci-symptomatic SARS-CoV-2 infection in a cohort of healthcare workers, (2) investigate the serological footprint of anti-S glycoprotein antibodies to HCoV-OC43, HCoV-HKU1, HCoV-229E, and HCoV-NL63, and determine whether pre-existing antibodies to these HCoVs are associated with altered COVID-19 disease course, and (3) determine if pre-existing immune responses to seasonal HCoV affect magnitude and duration of antibody and T cell responses to SARS-CoV-2 vaccination. The PASS study was initiated in late August of 2020 with a rolling enrollment period. The 3rd aim was added in December of 2020 following recognition that HCWs would be prioritized to receive newly available SARS-CoV-2 vaccines.

In addition to addressing the central aims, this study may also inform 1) the baseline frequency of adult individuals who have detectable antibodies to SARS-CoV-2 and other coronaviruses at the time of the study enrollment period, 2) the percentage of HCWs who seroconvert over a given time period, 3) identification of the most common symptoms that occur in individuals with pauci-symptomatic disease, 4) the magnitude and duration of SARS-CoV-2 antibody titers and T cell responses after seroconversion, 5) whether the presence of pre-existing antibody or T cells reactive to seasonal HCoVs alter magnitude or duration of SARS-CoV-2 antibody titers, 6) the longevity of detectable SARS-CoV-2 specific antibody and T-cell responses following SARS-CoV-2 vaccination, and 7) whether there are specific parameters of baseline immune function (innate or adaptive) associated with protection against clinical disease, development of local and/or systemic symptoms after vaccination, or robust antibody responses to vaccination. If study participants receive different SARS-CoV-2 vaccine formulations, this study may also provide information on possible differences in the magnitude and duration of antibody and T cell responses induced by each vaccine, as well as the relative effectiveness. Additionally, if substantial antigenic drift is observed in circulating strains of SARS-CoV-2, this long-term observational study may identify signals of immune enhancement upon subsequent wild-type viral exposure.

### Design overview and setting

PASS is a prospective and longitudinal observational cohort study on up to 300 healthcare workers at WRNMMC, a tertiary care hospital for active and retired military service members and their dependents in Bethesda, Maryland. An overview of the study design is depicted in Table 1.

**Table 1:** Schedule of study visits and sample collections

Clinic Visit Schedule	Baseline visit	Year 1	Year 2	Years 3 and 4
		Every month through August 2021	Every three months through August 2022	Every six months through August 2024
**Risk Exposure Questionnaire**	Monthly	Monthly	Quarterly ^b^	Semiannually
**Symptoms Review Questionnaire**	Baseline and daily if symptoms present	B-monthly, or daily if symptoms present	–	–
**Saliva for Coronavirus IgA**	1 × 0.3 ml	0–1 × 0.3 ml^a^	0–1 × 0.3 ml^a^	0–1 × 0.3 ml^a^
**Seurm Separator Tubes (SST) for CoVID multiplex serology analysis**	1 × 7.5 ml	1 × 7.5 ml	1 × 7.5 ml	1 × 7.5 ml
**Mononuclear Cell Preparation Tube (CPT Na Citrate or CPT Na heparin) for PBMCs and plasma isolation**	4–6 × 8 ml	0–6 × 8 ml^a^	0–6 × 8 ml^a^	0–6 × 8 ml^a^
**PAXgene for WB RNA transcriptonics**	1 × 3 ml	0–1 × 3 ml^a^	0–1 × 3 ml^a^	0–1 × 3 ml^a^
**WB in EDTA tube for DNA analysis of B cell and T cell repertoires**	1 × 2 ml	0–1 × 2 ml^a^	0–1 × 2 ml^a^	0–1 × 2 ml^a^
**Total blood volume (ml)**	44.5–60.5 ml	7.5 ml - 60.5 ml	7.5 ml - 60.5 ml	7.5 ml - 60.5 ml

^a^ Intermittent sample collections (e.g. at key timepoints after infection or vaccination)

The exact tubes to be drawn at each follow-up visit may change as determined by the primary investigator, but will not exceed 60.5 ml

**>** Study participants will visit the clinic once a month through August 2021, and then switch to quarterly and semiannual intervals as indicated below

^b^ Participants that become COVID-19 infected or vaccinated after April of 2021 may be asked to continue monthly visits for the first quarter of year 2

**>** Symptom questionnaires will end in August 2021

**>** Risk exposure questionnaires will continue quarterly or semiannually after August 2021

^**a**^ Details on alternative sample collections:

Participants that remain uninfected and unvaccinated -- > once yearly collection of saliva and all blood samples, except the PAXgene RNA tube

Participants that test COVID-19 positive by PCR, antigen, or antibody test

> first monthly visit after infection: collection of saliva and all blood samples, including EDTA and PAXgene RNA tubes

> visits 3 and 6 months after positive test, twice yearly in year 2, yearly in years 3 and 4: collection of saliva and all blood samples, except the PAXgene RNA

Participants vaccinated against COVID 19

> visits within 1 week of the first COVID-19 vaccination: collection of saliva and all blood samples, including EDTA and PAXgene RNA tubes

> visits within 1 week of the second COVID-19 vaccination: collection of saliva tube, 1x SST tube, 1x PAXgene RNA tube, and an EDTA tube

> visits 1, 6, and 12 months after final vaccination, twice yearly in year 2, yearly in years 3 and 4: collection of saliva and all blood samples, except the PAXgene

Study participants will additionally fill out monthly questionnaires regarding COVID-19 exposure risks, use of personal protective equipment (PPE), and social distancing practices (Attachment 7). At least twice monthly, participants will complete a standardized symptoms questionnaire (Attachment 6) validated for scoring viral respiratory disease severity. Should a participant develop any of the questionnaire symptoms, they will complete the questionnaire daily for as long as any of the symptoms persist. Additionally, participants will be asked to undergo SARS-CoV-2 PCR testing at the WRNMMC testing center any time they develop any symptoms consistent with COVID-19. Should any participant become seropositive for SARS-CoV-2 and/or obtain a positive SARS-CoV-2 PCR test, that individual’s cryopreserved baseline peripheral blood mononuclear cells (PBMCs) will be analyzed for T cell activation and cytokine production in response to peptide pools of SARS-CoV-2 proteins, and their NK cell numbers and functionality will be determined.

To enable longevity studies, after August of 2021 all enrolled participants will be followed quarterly during year 2 (through August of 2022) and then twice a year for the next 2 years (through August of 2024). Study participants will have blood and saliva samples obtained at each visit as detailed in the Schedule of Visits table. The monthly risk exposure/PPE/social distancing questionnaires will change to the amended schedule and the twice a month and on-demand symptoms questionnaires will stop after year 1. Any time study participants have symptoms consistent with COVID-19 they will be asked to continue to report to the WRNMMC COVID-19 testing center. Additionally, during years 2 through 4 study participants will be asked to contact the study clinic should they be diagnosed with COVID-19, at which point they will be sent an email link (Attachment 5) requesting they report peak symptom severity from home.

### Characteristics of participants and study recruitment

The goal of the PASS study is to enroll 300 SARS-CoV-2 seronegative healthcare workers, (HCWs) with no history of COVID-19, over a six-month time period recruited from Walter Reed National Military Medical Center (WRNMMC). Participants will be recruited through electronic media platforms and by distributing flyers in clinical and laboratory departments at WRNMMC. Recruitment will initially be conducted in areas of the hospital where HCWs have a high frequency of contact with confirmed or suspected cases of COVID-19 patients. These areas include the emergency department, COVID-19 outpatient testing center, COVID-19 inpatient wards, and the intensive care units. Study participants will be screened for eligibility as per the inclusion and exclusion criteria listed below. Because our goal is to evaluate immune responses in individuals not yet exposed to SARS-CoV-2, prior COVID-19 diagnosis as well as positive SARS-CoV-2 IgG response in our multiplex assay are exclusion criteria.

#### Inclusion criteria

- Age ≥ 18 years old
- Generally healthy
- Health care workers (nurses, doctors, physician assistants, respiratory therapists, patient transporters, medical intake personnel, occupational therapists, and medical technicians, and other related fields) at WRNMMC.
- Willingness and ability to return for scheduled follow-up visits, with assurance of follow-up for at least the first 3 months of study participation.

#### Exclusion criteria

- Immunocompromised state or immune modulating medications - presence of a disease that is actively causing severe immune suppression or active use of chronic immune modulating medications such as systemic corticosteroids at a dose equivalence of 20 mg prednisone or greater daily for over 1 month, chemotherapy, cytokine inhibitors, or agents that reduce T cell or B cell numbers or function
- Symptoms of fever, cough, anorexia, myalgias, chills, shortness of breath, anosmia, sore throat, rhinorrhea, or diarrhea at enrollment
- Presence of fever (T > 100.4 °F) on screening vital signs
- Known prior diagnosis with COVID-19
- Positive SARS-CoV-2 IgG by multiplex coronavirus serology assay
- Participation in a COVID-19 vaccine study or prophylactic antibody study

### Detailed study methods

During participant’s initial clinic visit informed consent (Attachments 1 and 2) and medical history (Attachment 3) will be obtained. Baseline samples will be collected and cryopreserved, to include: saliva for detection of CoV IgA and IgG, serum and plasma for analysis of CoV IgG and IgM levels, whole blood for DNA analysis of B and T cell repertoire (EDTA treated vacutainers) and RNA transcriptomic analysis (PAXgene RNA vacutainers), and isolation of baseline PBMCs. Additional data (Attachment 4) and sample collections will be performed at select study visits as detailed above (Table 1).

#### Symptoms questionnaire

REDCap-administered symptom questionnaires (FLU-PRO©) will be distributed daily in the form of a repeatable survey via email link (Attachment 5). Study participants will also be asked to complete the symptoms questionnaire at least twice a month during baseline periods of health and every day any symptoms are experienced. The symptoms questionnaire is based on a patient-reported outcome instrument that has been developed to standardize symptoms of respiratory viral infection in clinical research [30], and has been modified for COVID-19 by the addition of symptoms regarding loss of taste or smell. The index measures patient-reported severity and duration of 34 symptoms within the following symptom domains: nasal, throat, eyes, chest, gastrointestinal, body/systemic, and sense of taste/smell (Table 2). Severity of each symptom is measured on a scale of 0–4 (0 = not at all, 1 = a little bit, 2 = somewhat, 3 = quite a bit, and 4 = very much) for all symptoms except vomiting and diarrhea, which are scored in terms of frequency per day (0 = 0 times, 1 = 1 times, 2 = 2 times, 3 = 3 times, 4 = 4 or more times). Mean scores in each symptom domain are summed for a final daily symptom severity score of 0–28. This index can distinguish between asymptomatic, pauci-symptomatic, mild disease, moderate disease, and severe disease. An absolute difference of 3 points in total severity scores is hypothesized as considered clinically significant, and further analysis on clinical meaningfulness by domain and total scores will be conducted at the end of the study.

**Table 2:** Symptoms to be queried

**Nasal domain**	**Gastrointestinal domain**
• Runny or dripping nose	• Felt nauseated (like you wanted to vomit)
• Congested or stuffy nose	
• Sinus pressure	• Vomiting
• Sneezing	• Stomach ache
**Throat domain**	• Diarrhea
• Scratchy or itchy throat	**Body/Systemic domain**
• Sore or painful throat	• Felt dizzy
• Difficulty swallowing	• Head congestion
**Eye domain**	• Headache
• Teary or watery eyes	• Lack of appetite
• Sore or painful eyes	• Sleeping more than usual
• Eyes sensitive to light	• Body aches or pains
**Chest domain**	•Weak or tired
• Trouble breathing	• Chills or shivering
• Chest congestion	• Felt cold
• Chest tightness	• Felt hot
•Wet or loose cough	• Sweating
• Dry or hacking cough	**Sense domain**
• Coughing	• Loss of taste
• Coughed up mucus/phlegm	• Loss of smell

#### Risk exposure/PPE/social distancing questionnaire

Once per month study participants will complete an on-line REDCap questionnaire (Attachment 6) covering risk exposures, use of PPE, and social distancing practices (reminder emails will be sent to each participant monthly - Attachment 7). Each realm will be queried with regards to practices both at the hospital and away from the work setting. Specific questions for risk exposure at work include: number of days worked in the hospital in the last month, frequency of direct interaction with suspected or confirmed COVID-19 positive patients, and frequency of high risk hospital procedures such as intubation, airway suctioning, and administration of nebulizer therapy. Questions for home exposure risk include whether any members of a subject’s household have had symptoms consistent with COVID-19 or tested positive for COVID-19. PPE questions include frequency of PPE use in patient interactions, face mask use in the hospital when not interacting with patients, handwashing/hand sanitizer use before and after patient interactions, and use of a facemask outside of work. Responses will be based on a Likert scale and will include (1) none of the time, (2) less than half of the time, (3) about half of the time, (4) more than half of the time, and (5) all of the time. For the social distancing realm, the survey queries the number of times in the past month a subject has gone to the grocery store, eaten inside a restaurant, exercised in a public gym, taken public transit, attended a sporting event, and attended a public music, dance, or theatrical production. A final set of questions obtains information on the frequency of attending public gatherings with 1–2, 3–5, 6–10, 11–50, and > 50 people other than immediate members of an individual’s household.

#### Post-vaccination symptoms form

At the first monthly visit after receipt of a COVID-19 vaccine, study participants will complete a Vaccination Case Report questionnaire (Attachment 8) to report any symptoms they may have experienced post-vaccination. Each symptom is graded from 0 to 4 and duration of symptoms will also be noted.

#### Serum for CoV serologies

Serum will be collected monthly via Serum Separator Tubes (SSTs). Serological testing for IgM and IgG antibodies against prefusion stabilized SARS-CoV-2 S-2P glycoprotein ectodomain trimers [31–33]. hereafter referred to as spike protein, and HCoV spike proteins, will be will be run weekly on collected samples using an antigen-based multiplex microsphere immunoassay (MMIA) recently developed by members of our group (see assay details below) [14]. Positive results will be reported to participants as a research test result and will shift the scheduling of further blood draws as per Table 1. Excess samples will be stored at − 80 °C in 500 μl aliquots for subsequent analysis.

Analysis by the spike protein MMIA will enable simultaneous detection of IgG (or IgM or IgA) antibodies against the spike proteins of multiple seasonal and zoonotic coronaviruses, each of which is bound to a different microsphere. Each microsphere has a unique fluorescence signature, thus the platform allows for simultaneous measurement of antibody quantities for each of the medically-relevant CoVs. SARS-CoV-2, HCoV-229E and HCoV-NL63 spike proteins were procured from LakePharma, Inc. (Hampton, MA), and HCoV-OC43, HCoV-HKU1, MERS-CoV and SARS-CoV2 spike proteins were provided by Dr. Dominic Esposito (FNLCR, Frederick, MD) Spike proteins are coupled to carboxylated magnetic MagPlex microspheres (Bio-Rad, Hercules, CA) at a protein to microsphere ratio of 15 micrograms: 100 μl, and resuspended in a final volume of 650 μl following the manufacturer’s protocol (Bio-Rad) for amine coupling. Antigen-coupled microspheres are next loaded (in duplicate) into 96-well microtiter plates along with human serum samples tested at a 1:400 dilution in PBS and analyzed. Antigen-antibody complexes in each well are then washed with PBS-Tween20 (0.05%), incubated with secondary biotinylated anti-human IgG (or IgA or IgM), washed again, and then incubated with streptavidin-phycoerythrin. Antigen-antibody complexes are analyzed on Bio-Plex 200 multiplexing systems, and antibody levels are reported as a median fluorescence intensity (MFI). Threshold cutoffs for SARS-CoV-2 antibody positivity are set at 3 standard deviations above the mean MFI of archival serum samples collected in the U.S. between 2012 and 2018 and thus providing a naïve SARS-CoV-2 sera reactivity threshold. All serum samples will be simultaneously evaluated for antibodies against the spike proteins of SARS-CoV-2 as well as the four seasonal HCoVs. Post-SARS-CoV-2 seroconversion and post-vaccine samples will also be assessed for antibodies against the SARS-CoV-2 nucleocapsid protein (NP) enabling differentiation between infection and vaccination responses, and the SARS-CoV-2 RBD antigen (the immunodominant epitope of neutralization) to further evaluate the duration of antibodies that may have a role in neutralization. Finally, post-infection samples will also be evaluated for de novo development of antibodies against the SARS-CoV-2 related betacoronaviruses, SARS-CoV and MERS-CoV.

#### PCR testing

All participants enrolled in this study will be asked to be tested for active SARS-CoV-2 infection by nasopharyngeal swab PCR assay any and every time they develop any symptoms listed in Table 2. When any such symptoms develop, they are asked to report as soon as possible to the WRNMMC COVID-19 testing center. This facility is located directly in front of the WRNMMC Emergency Department entrance and has both walk-in and drive-through functionality. PCR tests are conducted in the hospital’s central diagnostic microbiology laboratory using a Roche Cobas 6800 system or a BioFire FilmArray Torch system, with a total capacity to run 996 tests per 24 h. Results of all SARS-CoV-2 PCR tests, as well as tests for any other respiratory tract infections, will be shared with the study team. Of note, it is a hospital requirement for WRNMMC Healthcare workers to obtain SARS-CoV-2 PCR testing whenever they are symptomatic. This requirement and the sharing of these test results with the study team, is included in the participant’s informed consent document for the PASS study.

#### Processing of other samples

PBMCs will be isolated from CPT tubes as per standard procedures and then cryopreserved in liquid nitrogen at 4 × 10^6^ cells/ml. Saliva samples (collected by the passive drool method), plasma and excess serum samples will be banked at − 80 °C. PAXgene RNA and EDTA vacutainers will be banked at − 20 °C. These samples will be maintained for subsequent analysis as dictated by study findings.

#### Disease severity information for study participants hospitalized with COVID-19

As we expect most participants who acquire SARS-CoV-2 infection during this study to not require hospitalization, comparative analyses evaluating disease severity will be based on peak symptom scores. In cases where study participants require hospitalization, we will also be collecting information from medical records on the following parameters: duration of hospitalization, duration of intensive care unit stay, daily oxygen saturation values, daily peak respiratory rates, results of chest imaging, placement on non-invasive ventilation (BIPAP), requirement for mechanical ventilation, and death.

#### Evaluation of baseline T cell responses

T-cell studies using cryopreserved PBMC samples obtained at baseline will be conducted on samples from all individuals diagnosed with COVID-19 (by seroconversion, positive PCR test, or positive antigen test) and on subsets of individuals who are vaccinated against COVID-19. We have chosen to use peptide pools for evaluating T cell responses because they are generally more sensitive than whole antigen for stimulation of cryopreserved PBMCs and the use of 15-mers enables peptide specific activation of both CD4^+^ and CD8^+^ T cells [34–37]. Additionally, the use of these pools will enable us to evaluate T cell responses against more of the variable (N-terminal) and conserved (C-terminal) regions of the SARS-CoV-2 S-glycoprotein. The peptide pools of the SARS-CoV-2 S-glycoprotein used by Braun et al. [38] are now commercially available from JPT Peptide Technologies and BEI Resources, as is a similar overlapping 15-mer peptide pool of the SARS-CoV-2 NP.

In this study we will measure the frequency of pre-existing T cell responses to the SARS-CoV-2 spike and NP antigens, as well as the cytokine profile produced by baseline PBMCs, after incubation with these pools. In order to conserve our cryopreserved PBMCs for possible future studies, we will measure both T cell activation and cytokine production using the same cell culture. Two million PBMCs will be incubated in 1 ml of media with either 1) the N-terminal SARS-CoV-2 S-glycoprotein peptide pool (JPT catalog #PM-WCPV-S-1 vial 1), 2) the C-terminal SARS-CoV-2 S-glycoprotein peptide pool (JPT catalog #PM-WCPV-S-1 vial 2), 3) the SARS-CoV-2 N protein peptide pool (JPT catalog #WCPV-NCAP-1), 4) the CMV pp65 peptide pool as a peptide positive control (JPT catalog #PM-PP65-2), 5) phytohemagglutinin (1mcg/ml) as a non-specific positive control, or 6) media plus DMSO (at a volume equal to that added with the peptide pools) as a negative control. Peptide pools will be used at a concentration of 1 mcg/ml (based on [38] and as per the manufacturer’s recommendation). Anti-CD28 antibody (clone CD28.2, BD Biosciences) will be added to each well at 1 mcg/ml for co-stimulation.

#### Measurement of cellular cytokine production in response to stimulation with peptide pools

Cells will be cultured for 24 h. Supernatants will be collected and measured for IL-2, − 4, − 5, − 6, − 10, − 13, −17A, − 21, TNFα, and IFNγ using a Luminex bead-based multiplex assay (R&D systems).

#### Measurement of activated CD4^+^ and CD8^+^ T cell frequencies by flow cytometry

Following supernatant collection for cytokine analysis, stimulation of cells will be stopped by addition of 20 mM EDTA in fresh media for 5 min. Cell will be collected and surface stained with anti-CD3 AF700 (BD Biosciences, clone sp34-2), anti-CD4 BV421 (BioLegend, clone OKT-4), anti-CD8 BUV395 (BD Biosciences, clone RPA-T8), anti-OX40/CD134 PE (BD Biosciences, ACT35), anti-4-1BB/CD137 APC (BioLegend, clone 4B4-1), and anti-CD69 APC-Fire750 (BioLegend, clone FN50), along with the addition of a viability dye (Zombie Green, BioLegend). Samples will be analyzed using a CytekAurora system. Activated CD4^+^ T cells will be identified as those that co-express 4-1BB and OX40, and activated CD8^+^ T cells as those that co-express 4-1BB and CD69 as per [39]. Use of these markers has been shown to be a sensitive approach for detecting frequencies of antigen-specific CD4^+^ and CD8^+^ T cells irrespective of T helper phenotype [39–41], and have been used successfully in studies of SARS-CoV-2 T cell responsiveness [39]. ELISPOT assays may also be conducted to enumerate numbers of IFNγ, IL-2, or other cytokine producing cells in response to various coronavirus peptide pools. Additionally, post-vaccination samples may be evaluated by flow cytometry for frequency of spike protein specific memory T cell subsets (effector memory, central memory, and stem cell memory) and potentially by ELISPOT for spike protein specific memory B cells.

#### Evaluation of frequency and functionality of NK cells and other innate immune cells

As with T cell studies, baseline NK cell analyses will be conducted on cryopreserved PBMC samples of individuals that seroconvert and/or test positive for SARS-CoV-2 by PCR or antigen test during the course of the study. Frequencies of NK cells will be determined by flow cytometry, with NK cells identified as immature (CD3^neg^CD56^bright^CD16^neg/dim^) or mature (CD3^neg^CD56^dim^CD16^positive^). NK cell function will be assessed by a target killing assay and CD107a expression. For the target killing assay, recently thawed PBMCs will be rested overnight in media with or without IL-2. Cells will then be incubated with K562 cells at NK cell to target cell ratios of 12:1, 6:1, 3:1, and 1.5:1. Target cell killing will then be evaluated using an NK-Target cell Visualization Assay (TVA) imaging platform available at the National Cancer Institute. Additionally, evidence of NK cell degranulation after incubation with target cells will be determined by flow cytometric staining for NK cell markers and CD107a.

Frequency and phenotype of innate immune cells (e.g. plasmacytoid dendritic cells, monocytes, and natural killer cells) may be performed on baseline samples and samples obtained after COVID-19 vaccination to obtain insight into their post-vaccination function and to determine whether correlates of robust antibody responses can be defined. Phenotyping will be conducted through multiparameter flow cytometry and Cellular Indexing of Transcriptomes and Epitopes by Sequencing (CITE-seq) of engaged innate immune cell populations responding to the SARS-CoV-2 vaccination.

#### Transcriptomics and DNA analyses

Blood samples in RNA stabilizing solution (PAXgene RNA tubes) and EDTA tubes will be stored frozen for potential future RNA or DNA sequencing. DNA samples will be banked for potential use in future studies evaluating genetic markers of resistance or susceptibility to severe disease with SARS-CoV-2. DNA sequencing may be conducted to determine changes in T cell and B cell repertoires after SARS-CoV-2 infection and/or vaccination.

#### Other immunological assays

Serum concentrations of chemokines, cytokines, vitamins and other molecules known to be important for immune cell function may be measured by ELISA or other standard assays.

#### Virtual meetings and/or electronic newsletters

To increase study participant satisfaction in being part of this natural history study, to enhance adherence to study protocol items such as regularly filling out on-line symptom and risk-exposure questionnaires, and to optimize long-term retention rates, we plan to hold periodic virtual update meetings and/or periodically distribute study update newsletters by email for PASS study participants.

The meetings and/or newsletters will review:

- The goals of the study
- Details of the protocol design
- Enrollment and retention numbers to date
- Presentation and discussion of any published study results.

The meetings may also include question and answer sessions so that participants can ask questions if they need clarification on any of the study protocols. Consent to be invited to virtual meetings and to receive electronic newsletters will be specifically asked for on a supplemental informed consent document (Attachment 2). In that document, participants will be notified that attendance at virtual meetings will result in their being identifiable by others.

### Power calculations and statistical analyses for primary study questions

#### Aim 1 primary questions

A. What percentage of Healthcare workers at WRNMMC become infected with SARS-CoV-2 over a 1 year period?
B. What percentage of participants acquiring SARS-CoV-2 infection develop no symptoms?

##### Sample size calculation

Confidence intervals of the percentages obtained will depend on both the infection rate and the percentage of individuals who develop asymptomatic infection. Three hundred study participants should be a sufficiently high enough number of individuals to perform descriptive statistical analyses of the percentages of individuals in the cohort who become infected with SARS-CoV-2 and who develop asymptomatic and/or pauci-symptomatic infection based on a 95% confidence interval for a proportion with a margin of error of 5 percentage points and up to 25% infection.

##### Statistical analyses

The infection rate will be determined by calculating the percentage of total study participants who become infected over a 1 year period. The numerator will be anyone meeting the case definition (SARS-CoV-2 IgG seroconversion or positive SARS-CoV-2 PCR or antigen test) and the denominator will be all individuals enrolled in the study with data from a 12 month encounter.

Asymptomatic individuals will be defined as those who exhibited no symptoms in the 4 weeks prior or 2 weeks after the timepoint of seroconversion or four-fold rise in SARS-CoV-2 antibody titer compared to baseline titers (or PCR diagnosis by a facility outside the research study). Division of this population by the total population in this study that become infected with SARS-CoV-2 will result in the percentage of individuals in this study who developed asymptomatic infection. 95% confidence interval will be determined using SPSS software.

Note: a similar analysis will be conducted for the percentage of asymptomatic plus pauci- symptomatic individuals, with pauci-symptomatic defined as individuals whose domain means on the symptoms questionnaire are below 1.5 (symptoms in each domain are scored from 0 to 4).

#### Aim 2 primary questions

A. What is the serological footprint of anti-S glycoprotein antibodies to HCoV-OC43, − HKU1, −229E, and -NL63?
B. Are pre-existing antibodies to these HCoVs associated with altered COVID-19 disease course?

##### Sample size calculations

Three hundred study participants should be a sufficiently high enough number of individuals to perform descriptive statistical analyses of the percentages of individuals in the cohort with detectable antibodies to each of the four major human coronaviruses, as well as the mean, range, and standard deviation for titers against each virus.

The sample size required for Aim 2 question B was determined using calculator A1 at the UCSF-CTSI sample size calculator website [42]. To conduct a study with a power of 0.8 and an alpha of 0.05 that can determine whether cross-reactive immune responses against SARS-CoV-2 are associated with more or less severe infection (as determined by a difference of 3.0 points on the symptom severity score and a standard deviation of 3.0 in each group) requires 17 participants per group. A cohort of 300 participants with a 20% dropout rate and a 25% infection rate would result in 60 infected individuals, and a 15% infection rate in 36 infected individuals.

##### Statistical analyses to be conducted

Aim 2 question A:

Descriptive statistical analyses will be performed to determine the percentages of individuals in the cohort with detectable antibodies to each of the four major human coronaviruses, as well as the mean, range, and standard deviation for titers against each virus.

Aim 2 question B:

Comparisons of SARS-CoV-2 symptom severity will be done on the basis of a T-test unless the data is not normally distributed, in which case we will use a Mann-Whitney test. The primary comparison will be made using peak severity scores of each individual during the 4 weeks prior and 2 weeks after seroconversion or four-fold increase in SARS-CoV-2 antibody titers (or PCR diagnosis made at a facility outside the research study).

We will also conduct regression models to adjust for age, gender, sex, and co-morbidities. We will examine the distribution of the data to choose an appropriate regression model, likely both linear and negative binomial.

NOTE: A cut-off for low and high titers will be determined prior to any statistical analysis. Alternatively, we may also perform correlation analysis with antibody titers and symptom severity index scores both treated as continuous variables.

#### Aim 3 primary question

##### Do pre-existing antibody titers against seasonal HCoVs affect the magnitude or duration of vaccine induced SARS-CoV-2 antibody titers?

To determine the sample size required to assess whether pre-existing antibody levels against the seasonal HCoVs are associated with peak levels of vaccine-induced SARS-CoV-2 specific antibody responses, we utilized the University of California at San Francisco Sample Size Calculator for Correlations [42]. A sample size of 194 participants is required to be able to detect a correlation (rho factor) of 0.2 with a power of 0.80 and an alpha value of 0.05. Given our goal cohort size of 300 study participants, the study will be powered to detect a correlation of 0.2 even if we have 30% dropout. Of note, should we have 50% dropout, then we would still be powered to detect a correlation of 0.3 (which, for a power of 0.8 and an alpha of 0.05, only requires 85 total participants).

Linear regression and correlation analyses will be conducted to determine if antibody levels against any of the seasonal HCoVs are associated with peak levels of vaccine-induced SARS-CoV-2 antigen-specific antibody responses. We will also conduct regression models to adjust for potential confounding variables such as age and sex.

Cox proportional hazards and/or Kaplan Meier analyses will be conducted to determine if antibody levels against any of the seasonal HCoVs are associated with differences in duration of detectable levels of SARS-CoV-2 specific antibody responses. Cox proportional hazards regression models will also be conducted to adjust for potential confounding variables such as age and sex.

Note: Description of secondary study questions can be found in the Supplemental file - S1. Secondary study questions with planned statistical analysis.docx.

## Discussion

The newly emerged SARS-CoV-2 virus, the causative agent of the COVID-19 pandemic, can elicit a range of symptoms from asymptomatic to severe respiratory illness. In this prospective assessment of healthcare workers our principal goals are to document the frequency of asymptomatic and pauci-symptomatic disease within this at-risk cohort, and to determine whether pre-existing antibodies and/or T cells responses specific for seasonal HCoVs play a role in the immunological or clinical responses to SARS-CoV-2 infection or vaccination.

By collecting monthly serum samples from study participants, we will be able to catch variations in seasonal HCoV antibody repertoires that are likely to be altered by seasonal exposures. An added benefit from this study will be insights gained on the baseline prevalence of seasonal HCoV antibodies in this adult population, the frequency of seasonal HCoVs infections, and persistence of relevant antibody titers over time. We expect the majority of study participants will have detectable IgG antibodies against the spike proteins of all four major seasonal HCoVs at baseline and that the relative levels of these antibodies will display wide variability. We predict that high baseline titers of antibodies against HCoVs will be associated with lower symptom severity.

In addition to monthly serum samples we will have cryopreserved donor-specific baseline PBMCs for the determination of pre-existing SARS-CoV-2 cross-reactive T cells. PBMCs from individuals that develop COVID-19 infection in this study (defined by seroconversion or positive PCR or antigen testing) will be analyzed for responsiveness to peptides from the variable N-terminal and conserved C-terminal regions of the SARS-CoV-2 S glycoprotein, as well as to the SARS-CoV-2 NP. PBMC samples will also be analyzed for NK cell number and functionality. We predict that few individuals will have baseline T cell responses to peptides from the variable N-terminal region of the SARS-CoV-2 S glycoprotein, and that one third of individuals will have measurable T cell responses to peptides from the more conserved C-terminal region and to peptides from the NP based on prior studies of T cell responses in uninfected individuals [43, 44]. Further, we predict that presence of cross-reactive T cell responses will be associated with milder disease, except when the baseline T cell response demonstrates a predominant Th2 (IL-4, IL-5, and IL13) cytokine response to any of the SARS-CoV-2 peptide pools. We predict that low frequencies and/or decreased function of NK cells will be associated with more severe disease. If cell numbers permit, additional phenotypic and functional studies of the myeloid and B cell compartments can also be conducted and compared to T and NK cell responses.

In the third aim, we will study the magnitude and longevity of immune responses post-COVID-19 vaccination in study participants. Using baseline PBMCs as well as PBMCs obtained during the first week after vaccination, we should be able to identify innate and/or adaptive immune responses that may be associated with robust COVID-19 vaccine responses. We will also be able to determine whether pre-existing antibody and/or T cell responses to the seasonal coronaviruses have an effect on vaccine-induced antibody levels or duration. Additionally, we will be able to characterize the magnitude and longevity of antibody and T cell responses of COVID-19 vaccination in an adult cohort of healthcare workers, potentially providing important information on whether and when individuals may need repeat vaccination.

Conducting a prospective natural history study in which uninfected individuals are followed over time does have a number of limitations. The greatest potential limitation of this study design is that it may not capture sufficient numbers of infections to determine the effects that baseline immune responses may have on clinical and immune responses to SARS-CoV-2. That stated, a seroconversion rate as low as 15% over the course of the next year should enable sufficient power for us to achieve our Aim 2 goals, even if we experience 20% dropout. If seroconversion rates are very low, then results of this study could potentially be combined with those of other prospective, longitudinal cohort studies of SARS-CoV-2 infection, especially as we are cryopreserving serum, plasma, and PBMCs at baseline to be used as needed for future studies.

Another challenge for this study will be retaining study participants with monthly clinic visits and symptom questionnaires every day they have symptoms (plus at least twice a month when they have no symptoms) over a 1 year period. We have taken several steps to mitigate dropout. These include: compensating participants for each study clinic visit and for regularly completing symptom questionnaires, having blood collections take place at a clinic that is located within a short walk from the main hospital, and accounting for up to a 20% dropout rate in the first year. Additionally, we plan to hold periodic virtual meetings with study participants in which investigators will review the goals of the study and provide updates on any published findings from the study. In addition to potentially increasing subject adherence to the study protocols (such as regularly filling out symptom and risk exposure questionnaires and being tested for COVID-19 whenever one is symptomatic), we hope that these meetings will provide study participants with a sense of communal purpose with regards to continuing participation in the study.

A final limitation with this type of natural history study is with regard to our ability to make comparisons between different COVID-19 vaccinations or vaccine schedules, or to evaluate the long-term efficacy of COVID-19 vaccination in those individuals that receive them. This study will not attempt to influence individuals on which vaccine they receive (which is dependent on what our hospital system is able to offer), nor whether they are in fact vaccinated at all (which is currently a recommendation from WRNMMC, but not a requirement). Thus, we have included comparisons of immune responses and clinical efficacy by different COVID-19 vaccines and vaccine schedules as possible secondary questions the study may be able to address, dependent on vaccine uptake in the cohort.

In addition to its inherent limitations, this prospective natural history design also has certain advantages to other study designs. While many groups have established repositories of PBMCs and serum obtained from participants during and after SARS-CoV-2 infection, very few have pre-infection samples. While such post-diagnosis studies can compare immune responses in those with mild versus severe infection, conclusions regarding differences in immune responses are limited in their ability to assign causality. In other words, it is unclear from post-infection studies whether differences in immune responses after infection are the cause of or due to severe disease. Further, retrospective analyses in which individuals use archived serum samples are limited by the fact that the samples are often obtained from timepoints quite distant from time of infection. By conducting this study in a prospective fashion in which PBMCs and serum samples are obtained at baseline followed by monthly collection of serum, this study has the potential to more clearly inform whether there are specific pre-existing immune factors that are important for shaping the clinical and immune responses to both SARS-CoV-2 infection and vaccination.

This study is also innovative in that the multiplex array we have developed is a unique and cutting-edge tool to measure specific and cross-reactive antibody responses against the S-glycoproteins of SARS-CoV-2, and HCoV-OC43, HCoV-HKU1, HCoV-229E, and HCoV-NL63 (as well as SARS-CoV, MERS-CoV). In using this assay, we will be one of the first to provide robust information on the prevalence and magnitude of pre-existing antibody responses to the S glycoproteins of all four major HCoVs. Additionally, this study may provide insight to potential protective or detrimental contributions of pre-existing HCoV antibodies and cross-reactive T cells to COVID-19 disease severity and vaccine-induced immune responses.

While it is clear that cross-reactive immune responses occur, their clinical significance remains unknown. Consequently, serology data generated in this study can potentially inform rational vaccine design programs and multivalent vaccine platforms aimed at developing a pan-coronavirus vaccine structural candidate. Taken together, findings from this study will have implications for vaccine design, not just for SARS-CoV-2, but for potential future novel CoVs as well. This study may also provide insights on whether certain baseline immunological factors can be used to risk stratify individuals as high or low for severe COVID-19 disease or weak immune response to COVID-19 vaccination.

## Supplementary Information

**Additional file 1. Supplemental file S1.** Secondary study questions.

**Additional file 2.** Informed consent form.

**Additional file 3.** Informed consent addendum.

**Additional file 4.** Medical intake form.

**Additional file 5.** Monthly CTC visit questionnaire.

**Additional file 6.** Email reminder - symptoms questionnaire.

**Additional file 7.** Risk exposure questionnaire.

**Additional file 8.** Email reminder - risk exposure questionnaire.

**Additional file 9.** COVID-19 vaccination history form.

## Data Availability

Not applicable - the purpose of this article is to define the intent and experimental design of the Prospective Assessment of SARS-CoV-2 Seroconversion (PASS) study.

## Abbreviations

ADE: Antibody-dependent enhancement
BIPAP: Bilevel Positive Airway Pressure
CITE-seq: Cellular Indexing of Transcriptomes and Epitopes by Sequencing
CoV: Coronavirus
COVID-19: Coronavirus Disease 2019
HCoV: Human coronavirus
HCWs: Healthcare workers
IgA: Immunoglobulin A
IgG: Immunoglobulin G
IgM: Immunoglobulin M
MFI: Median fluorescence intensity
MMIA: Multiplex Microsphere Immunoassay
NK cell: Natural killer cell
NP: Coronavirus Nucleocapsid protein
PASS: Prospective Assessment of SARS-CoV-2 Seroconversion Study
PBMCs: Peripheral Blood Mononuclear Cells
PCR: Polymerase chain reaction
PPE: Personal protective equipment
S protein: Coronavirus spike protein
TVA: Target cell Visualization Assay
WRNMMC: Walter Reed National Military Medical Center
