# Supplementary material for: Prospective Assessment of SARS-CoV-2 Seroconversion (PASS) study: an observational cohort study of SARS-CoV-2 infection and vaccination in healthcare workers
Source: BMC Infect Dis. 2021 Jun 9;21:544. doi: 10.1186/s12879-021-06233-1 (PMC8188741; doi:10.1186/s12879-021-06233-1)
Supplement: Supplementary file 1 — Additional file 1. Supplemental file S1. Secondary study questions. [file 12879_2021_6233_MOESM1_ESM.docx]

**Secondary study questions with planned statistical analysis**

**Are there baseline differences in innate or adaptive immune function between individuals who develop asymptomatic or pauci-symptomatic infection with those that develop more severe clinical disease?**

We will examine the relationship between various factors of immune response (frequencies of innate immune cell subsets, basal activation status of innate immune cell subsets, basal expression of pattern recognition receptors that control RNA viruses, baseline concentrations of vitamins, cytokines, and other factors known to affect immune system function, presence of any adaptive immune responses to SARS-CoV-2 at baseline, functionality of NK cells at baseline, initial skewing of CD4^+^ T helper response at baseline, etc.) with symptom severity score. We will develop regression models with the outcome of symptom severity on the chosen function of the immune response to see if there is a statistically significant relationship. Covariates to be included in the regression analyses include confounders such as specific co-morbidities, age, sex, race, medications used, etc.

**Do pre-existing antibody or T cell responses alter magnitude or duration of SARS-CoV-2 antibody titers after natural infection?**

Linear regression analyses will be conducted to determine if antibody levels against any of the seasonal HCoVs or baseline T-cell responses against SARS-CoV-2 are associated with greater peak antibody level or increased duration of SARS-CoV-2 specific antibody responses.

**What happens to the magnitude and duration of antibody titers against seasonal coronaviruses?**

Descriptive statistics (including average, median, and standard deviation) will be used to describe the peak titer levels and duration of detectable antibody titers against the seasonal HCoVs.

## What happens to the magnitude and duration of SARS-CoV-2 titers over time?

Descriptive statistics (including average, median, and standard deviation) will be used to describe the peak titer levels and duration of detectable SARS-CoV-2 titers over time in individuals who seroconvert.

**Identification of risk factors for development of infection**

Descriptive analyses will be performed to describe trends in use of PPE and social distancing over the course of the outbreak. Logistic regression analyses with repeated measures will be conducted to determine if variables such as risk exposures and use of PPE and social distancing are associated with development of SARS-CoV-2 infection.

**Do pre-existing T cell responses against SARS-CoV-2 affect the magnitude or duration of vaccine-induced SARS-CoV-2 antibody titers or duration?**

Levels and duration of vaccine-induced SARS-CoV-2 vaccine responses will be compared between individuals that have measurable pre-existing T cell responses to SARS-CoV-2 peptides to those that do not on the basis of a T-test unless the data is not normally distributed, in which case we will use a Mann-Whitney test. We may also conduct regression models to adjust for potential confounding variables such as age and sex.

**Assessment of different COVID-19 vaccines and vaccine schedules**

If study participants receive different COVID-19 vaccines, or different schedules of the same vaccines (such as one versus two doses of a two-dose vaccine, or as recommended versus more prolonged timing between first dose and booster) then comparisons of peak vaccine-induced antibody titers and duration of antibody responses will be made on the basis of a T-test unless the data is not normally distributed, in which case we will use a Mann-Whitney test. If the cohort receives three or more different COVID-19 vaccines, then comparisons will be made using analysis of variance. Additionally, linear regression models and Cox proportional hazards regression models will be conducted to adjust for potential confounding variables with regard to peak titers and duration of antibody responses, respectively.

**Assessment of the magnitude, duration, and quality of antibody and T cell responses induced by COVID-19 vaccination**

Descriptive statistics (including average, median, and standard deviation) will be used to describe peak SARS-CoV-2 antibody titers, duration of detectable SARS-CoV-2 titers, duration of neutralizing antibody titers to SARS-CoV-2, and the percentage of T cell responses that are Th1 or Th2 skewed in individuals that are COVID-19 vaccinated. Additionally, linear regression models and Cox proportional hazards regression models will be conducted to adjust for potential confounding variables with regards to peak titers and duration of antibody responses, respectively.

**Determine whether vaccine-induced adverse effects correlate with the magnitude of vaccine-induced antibody levels**

Magnitude and duration of COVID-19 vaccine-induced antibody titers and T-cell responses will be evaluated for correlation with vaccine-induced symptom scores (as documented on the COVID-19 vaccine history form) on the basis of Spearman rank analysis. Regression modeling will be conducted to adjust for potential confounders such as age and sex.

**Assessment of protective efficacy of COVID-19 vaccines**

We anticipate most participants will elect to be vaccinated. However, if substantial numbers do not get vaccinated, then longitudinal assessment of the cohort may enable assessment of vaccine efficacy over time. If a substantial percentage of the cohort remains unvaccinated, then comparisons of the frequency with which COVID-19 infections occur in vaccinated versus unvaccinated populations will be made on the basis of a T-test unless the data is not normally distributed, in which case we will use a Mann-Whitney test.

**Effect of prior COVID-19 infection on vaccine-induced antibody and T cell responses**

Levels and duration of vaccine-induced SARS-CoV-2 antibody responses will be compared between individuals that were and were not previously infected with COVID-19 on the basis of a T-test unless the data is not normally distributed, in which case we will use a Mann-Whitney test. We may also conduct regression models to adjust for potential confounding variables such as age and sex.

**Correlations between innate immune responses present at baseline and one week after vaccination with magnitude and duration of vaccine-induced antibody responses**

Linear regression models and Cox proportional hazards regression may be conducted to determine if baseline frequencies or phenotypes of innate immune cells (NK cells, plasmacytoid dendritic cells, or monocytes), or activation status of these innate immune cells within one week of first vaccination, are associated with differences in vaccine-induced antibody titers or duration. Cox proportional hazards regression models will also be conducted to adjust for potential confounding variables such as age and sex.

**Identification of adaptive immune factors present at baseline that are associated with magnitude and/or duration of vaccine-induced antibody responses**

We will examine the relationship between various factors of adaptive immune response with magnitude and duration of vaccine-induced antibody responses. We will develop regression models with the outcome of symptom severity on the chosen function of the immune response to see if there is a statistically significant relationship. Covariates to be included in the regression analyses include confounders such as specific co-morbidities, age, sex, and race.

**Correlations between vitamin levels present at baseline and inflammatory markers present after vaccination**

Linear regression models may be conducted to determine if baseline concentrations of vitamins known to be important for immune function (such as vitamins D, A, and C) are associated with changes in concentrations of inflammatory cytokines or frequencies of activated innate immune cells after COVID-19 vaccination. Regression models will also be conducted to adjust for potential confounding variables such as age and sex.

**Assessment of baseline immune functions, and local and systemic symptoms to COVID-19 vaccination**

Linear regression models may be conducted to determine if baseline antibody levels against the seasonal coronaviruses, baseline cross-reactive T cell responses to SARS-CoV-2 spike protein, or baseline NK cell numbers or subtypes are associated with differences in severity of local and systemic reactions to COVID-19 vaccination. Regression models will also be conducted to adjust for potential confounding variables such as age and sex.
