# Supplementary material for: Prospective Assessment of SARS-CoV-2 Seroconversion (PASS) study: an observational cohort study of SARS-CoV-2 infection and vaccination in healthcare workers
Source: BMC Infect Dis. 2021 Jun 9;21:544. doi: 10.1186/s12879-021-06233-1 (PMC8188741; doi:10.1186/s12879-021-06233-1)
Supplement: Supplementary file 2 — Additional file 2. Informed consent form. [file 12879_2021_6233_MOESM2_ESM.pdf]

Meets 2018 Common Rule Requirements

Naval Medical Research Center Clinical Trial Center (NMRC CTC)
CONSENT TO PARTICIPATE IN RESEARCH
Title: *Prospective Assessment of SARS-CoV-2 Seroconversion (PASS)*
Principal Investigator: [redacted]

You may be eligible to take part in this research study. This form gives you important information about the study.

Please take time to carefully review this information. You should talk to the researchers about the research study and ask them any questions you have. You may also wish to talk to others (for example, your friends, family, or your personal physician) about your potential participation in this research study. You do not have to take part in this study. Participation is voluntary. You may also leave the research study at any time without penalization.

1. KEY INFORMATION:

You are invited to take part in an observational research study of the 2019 novel coronavirus disease (COVID-19 also known as SARS-CoV-2 infection) to learn more about this disease and how we can possibly better manage and treat it. Taking part in this research is entirely voluntary. If you choose not to participate in this research study, your decision will not affect your eligibility for care or any other benefits to which you are entitled. Please read the information below, and ask questions about anything you do not understand, before deciding whether or not to take part in the study.

The goal of this study is to better understand how common SARS-CoV-2 infection is among healthcare workers, clinical presentation, and immune responses in healthcare workers at the Walter Reed National Military Medical Center (WRNMMC) over the course of up to four years. We also want to determine if previous exposure to seasonal coronaviruses or other aspects of baseline immune system function affects the duration or strength of protective immune responses you get from SARS-CoV-2 vaccination.

If you choose to participate:
(1) Until August of 2021, you will be asked to complete an online symptom questionnaire twice a month (and daily when symptoms are present), an online risk exposure/PPE use/social distancing questionnaire once a month, and to have blood drawn and possibly saliva collected once a month. Blood and saliva specimens will be tested for immune responses and measurement of antibodies to SARS-CoV-2 and other coronaviruses. The primary study procedures will be the monthly blood draws and possible saliva collection (for which you will be asked to passively drool into a sample collection tube for 5-10 minutes).

Please note that the investigators understand that it can be challenging to return to the study clinic every month. Study subjects are welcome to continue to be followed in the study even if they are unable to make it to all monthly visits.

(2) All study subjects will be followed once a month until August, 2021. At that point, you will change to being seen quarterly (about once every three months) in the clinic. Subjects that are vaccinated against COVID-19 or diagnosed with SARS-CoV-2 infection from May to August of 2021 may be asked to continue monthly clinic visits for an additional three months before switching to quarterly visits. Subjects remaining in the study during years 3 and 4 will be asked to return to the clinic just twice a year.

(3) Study subjects that receive a COVID-19 vaccine will be asked to fill out a COVID-19 vaccine questionnaire after each vaccination.

(4) After August of 2021, symptom questionnaires will stop.

(5) After August of 2021, we will ask you to complete a risk exposure/PPE use/social distancing questionnaire once every three months during year 2, and then twice a year for years 3 and 4.

(6) Study subjects are asked to report to the WRNMMC COVID-19 testing center whenever experiencing symptoms consistent with COVID-19.

Some of the questions the study hopes to answer include:

1) What percentage of healthcare workers at WRNMMC already have antibodies against SARS-CoV-2 and other human coronaviruses?
2) Over the course of the next year, what percentage of healthcare workers develop infection, and of those that do, what percentage will have no symptoms or only mild symptoms?
3) What are the most common symptoms of mild infection?
4) Is the presence of pre-existing antibodies, cross-reactive T-cells, or other immune responses, associated with more or less severe disease?
5) How long do antibody and cellular immune responses to SARS-CoV-2 infection last?
6) How do baseline immune function and previous exposure to seasonal coronaviruses affect the protection you would get from SARS-CoV-2 vaccination?

Our hope is that information learned from this study can be used to improve strategies to control this pandemic.

If you decide to take part in this research study, you will be asked to sign this document. Before you sign this document, be sure you understand what the research study is about in all sections of the consent form, including the risks and possible benefits to you.

2. WHAT IS THE PURPOSE AND DURATION OF THIS RESEARCH AND WHO WILL TAKE PART?

You are being asked to take part in this research study because you are either

A) a healthcare provider at the WRNMMC and are willing and able to return for follow-up visits at least for the first three months of the study,

OR

B) a worker at the Uniformed Services University (USU) willing to provide blood and/or saliva primarily for use in optimization of the research assays being conducted in this study.

The purpose of this research study is to learn about the prevalence of the 2019 novel coronavirus disease (COVID-19) (how common is the disease), the clinical presentation (symptoms associated with the disease), and the immune responses (how your body recognizes and defends itself against the virus) in healthcare workers at WRNMMC over the course of one year. In addition, we also want to determine if baseline immune function or previous exposure to seasonal coronaviruses affects the protection one would get from SARS-CoV-2 vaccination, more specifically the strength and duration of protection in healthcare workers at WRNMMC over the course of three years after the final COVID-19 vaccination.

For study subjects, the duration of participation per visit will be about one hour per session for one visit a month until August of 2021. In year two, you will have about four visits, and in years 3 and 4 of the study you will have two visits per year.

We plan to enroll up to 300 healthcare workers for the study, as well as an additional 20 Uniformed Services University affiliated individuals to serve as blood donors for the purpose of optimizing research tests.

<u>For WRNMMC healthcare workers:</u>
There will be up to 300 healthcare workers taking part in the study at the Naval Medical Research Center Clinical Trials Center (NMRC CTC) over a period of 48 months.

During the first year of the study, you will have one visit a month until August of 2021 with an NMRC CTC research staff member. In year two, you will have about four visits, and in years 3 and 4 of the study you will have two visits per year.

<u>For USU affiliated individuals:</u>
There will be about 20 USU affiliated individuals taking part in the study at the NMRC CTC. Your blood and/or saliva will be used to optimize the immunological assays to be conducted in this study. *You will not have regular monthly clinic visits, and you will not be asked to fill out the symptom or risk exposure questionnaires.* You may be asked to return to the clinic to provide additional blood and saliva samples up to once a month as needed.

Subject Initials:___________(initial here to acknowledge that you are a USU affiliated individual)

<u>For all study participants:</u>
During the study, you will be informed if your blood tests positive for either IgG or IgM antibodies against SARS-CoV-2. The antibody tests we will be using have been developed for research purposes and are not FDA-approved. A positive test result may be an indicator of recent infection with SARS-CoV-2, the virus that causes COVID-19 disease. These tests do not indicate whether a person has an active infection, or whether a person is protected from future infection.

Throughout the study period, all subjects with <u>any symptoms</u> consistent with COVID-19 will be asked, consistent with WRNMMC policy, to immediately wear a face mask and be evaluated for SARS-CoV-2 infection and PCR testing at the WRNMMC testing center or through their primary healthcare provider.

If your IgG antibody test turns positive, you will be contacted by a PASS team staff member and asked if you have had any recent symptoms consistent with COVID-19. If you have had recent symptoms

and have not yet been evaluated for SARS-CoV-2 infection, you will be asked to don a mask and be evaluated for SARS-CoV-2 infection at either WRNMMC or by your primary healthcare provider. If your IgG antibody test is positive but you have had no symptoms in the past two weeks consistent with COVID-19, then you will be advised on whether to obtain further testing based on the most up to date CDC recommendations.

If your SARS-CoV-2 IgG antibody test is negative but your IgM antibody test is positive, then you will be asked to wear a mask and you will be referred to the WRNMMC SARS-CoV-2 testing center to be tested for active infection regardless of the presence or absence of symptoms, as a positive IgM test with a negative IgG test may be indicative of a very recent exposure.

Additionally, you will be asked at every clinic visit to share the results of any COVID-19 tests that have been conducted on yourself outside this research study since the last clinic visit, as well as about any COVID-19 vaccinations or other vaccinations you may have received. Other than any positive blood SARS-CoV-2 antibody tests, the research results about you will not be shared with you. Published papers based on the results of this study will be shared with study subjects by email.

3. SCREENING PROCESS TO QUALIFY FOR PARTICIPATION IN THIS STUDY

If you consent to participate in this study, then after signing this informed consent document you will need to provide some information so that the NMRC CTC staff can confirm that you qualify for the study. This is called the "Screening Process". Research staff will determine if you can participate based on your responses on a medical intake form and serological tests. In situations where your medical history is unclear, study staff may review your medical records to confirm that you may participate.

4. WHAT WILL HAPPEN IF YOU DECIDE TO BE IN THIS RESEARCH?

For healthcare workers participating in this study:
Your medical records may be reviewed now and in the future for information related to COVID- 19 and concurrent and past conditions and medications. We will ask you about your health and will collect blood and saliva for research testing purposes.

At home you will complete the following study procedures:

- Complete a symptom questionnaire at least twice a month (will take about 5-8 minutes to complete). If you have symptoms consistent with a respiratory virus infection, please complete every day you are symptomatic. These include:

1. Runny or drippy nose
2. Congested or stuffy nose
3. Sinus pressure
4. Scratchy or itchy throat
5. Sore or painful throat
6. Difficulty swallowing
7. Teary or watery eyes
8. Sore or painful eyes
9. Eyes sensitive to light
10. Trouble breathing
11. Chest congestion
12. Chest tightness
13. Wet or loose cough
14. Dry or hacking cough
15. Feeling nauseous (like you want to throw up)
16. Stomachache
17. Feeling dizzy
18. Head congestion
19. Headache
20. Lack of appetite
21. Sleeping more than usual
22. Body aches or pains
23. Weak or tired
24. Chills or shivering
25. Feeling cold
26. Feeling hot
27. Sweating
28. Loss of taste
29. Loss of smell

- Report to the WRNMMC COVID-19 Testing Center to be tested for COVID-19 every time you develop any of the above symptoms.
- Complete a risk-exposure, use of personal protection equipment (PPE), and social distancing questionnaire once a month (will take about 5-8 minutes to complete).

At each study visit, once a month until August of 2021, and then at more spaced out intervals in subsequent years, you will complete the following study procedures:

- Discuss with the clinic team any issues with filling out the on-line questionnaires, notify the team if you have been tested for COVID-19 since the last visit and/or if you have received a COVID-19 vaccination, and update the team with regards to any major changes to your health or medication use since the last visit
- Possibly provide saliva for SARS-CoV-2 antibody testing
- Provide blood samples for SARS-CoV-2 antibody testing and other immunological tests (intake visit of blood: 7 tubes, approximately 44.5 ml or 3.0 tablespoons; monthly visits: 1 tube, approximately 7.5 ml or about half a tablespoon). Note: Up to an additional 7 tubes of blood may be drawn at the regular monthly visits, increasing the total potential number of blood tubes drawn at the regular monthly visits to 8 tubes (maximum 58.5 ml, about 4 tablespoons).
- A summary of the research study timeline with expected blood and saliva collections is shown in the table below.

Schedule of Visits and Samples to be Obtained

- All study subjects will be followed in the clinic once a month through August 2021, and then switched to more spaced out visits
- Symptom questionnaires will end in August 2021
- Risk exposure questionnaires will continue quarterly or semiannually after August 2021

		Year 1	Year 2	Years 3 and 4
**Clinic Visit Schedule**	**Baseline Visit**	**Every month through August 2021**	**Every three months through August 2022**	**Every six months through August 2024**
**Risk Exposure Questionnaire**	Monthly	Monthly	Quarterly*	Semiannually
**Symptoms Review Questionnaire**	Yes and daily if symptoms present	Yes and daily if symptoms present	No	No
**Saliva for Coronavirus IgA**	Yes (one tube)	0-1 tubes	0-1 tubes	0-1 tubes
**Serum Separator Tubes (SST) for COVID multiplex serology analysis**	1 x 7.5ml	1 x 7.5ml	1 x 7.5ml	1 x 7.5ml
**Mononuclear Cell Preparation Tube (CPT Na Citrate or CPT Na Heparin) for PBMCs and plasma isolation**	4-6 x 8ml	0-6 x 8ml	0-6 x 8ml	0-6 x 8ml
**PAXgene for WB RNA transcriptomics**	1 x 3ml	0-1 x 3ml	0-1 x 3ml	0-1 x 3ml
**WB in EDTA tube for DNA analysis of B cell and T cell repertoires**	1 x 2ml	0-1 x 2ml	0-1 x 2ml	0-1 x 2ml
**Total blood volume (ml)**	44.5ml - 60.5ml	7.5ml - 60.5ml	7.5ml - 60.5ml	7.5ml - 60.5ml

* Subjects that become COVID-19 infected or vaccinated after April of 2021 may be asked to continue monthly visits for the first quarter of year 2

Boxes in white --> samples we expect to always obtain at each of these timepoints
Boxes in grey --> what we expect to obtain intermittently (especially at key timepoints after vaccination or infection)

<u>Details on when we anticipate drawing a full set of saliva and blood samples (grey boxes)</u>

- For individuals that remain uninfected and unvaccinated --> once yearly draw all blood and saliva tubes except for the PAXgene tube
- For individuals that test COVID-19 positive by PCR, antigen, or antibody test

  - At first monthly visit after infection draw all blood and saliva tubes, including both the EDTA and PAXgene tubes.
  - At visits at 3 and 6 months after positive test, twice yearly in year 2, and yearly in years 3 and 4 draw all blood and saliva tubes except the PAXgene tube.
- For all individuals vaccinated against COVID 19
  - At visits 1, 6, and 12 months after final vaccination, twice yearly in year 2, and yearly in years 3 and 4 draw all blood and saliva tubes except the PAXgene tube
- <u>Individuals that come to clinic within one week of their first COVID-19 vaccination</u> draw a full set of blood and saliva tubes, including both the PAXgene and EDTA tubes
- <u>Individuals that come to clinic within one week of their second COVID-19 vaccination</u> draw a saliva tube, an SST tube, a PAXGene tube, and an EDTA tube

Note: The exact tubes to be drawn at each follow-up visit may change as determined by the primary investigator, but will not exceed 60.5 ml.

<u>For USU affiliated workers participating in this study to provide blood samples for assay optimization:</u>

At the first visit, saliva and or blood will be collected. Total blood volume will be from 1 tube (7 ml, to a maximum of 8 tubes, 58.5 ml). After the first visit, you will be contacted by phone or email should additional clinic visits for saliva or blood samples be required.

5. <u>WHAT ARE THE RISKS OR DISCOMFORTS FROM BEING IN THIS RESEARCH?</u>

Being a part of this study means that blood and saliva samples will be taken for research purposes. There is a risk of pain or irritation when samples are taken. Blood draws entail a risk of bleeding, pain, bruising, or rarely, fainting or infection at the blood draw site. However, these risks are not different from when you donate blood or have blood collected by your healthcare provider. To minimize the risk, only experienced staff will draw blood in this study.

As part of the blood testing, we will be obtaining DNA and RNA. DNA and RNA testing to be done will be focused on changes in the immune response after positive antibody testing and may also be done to evaluate for genetic differences that may predispose an individual to more severe disease.

When DNA testing is done, there is a small chance that the results will show a genetic condition that could affect your future health. Since the tests will be coded, no one on the study team will know that such a result applies to you, and in any case there is usually considerable uncertainty about the implications of any research DNA test result for individual health. For these reasons we would not attempt to identify you or inform you of any results from DNA testing.

Pregnant women can be a part of this study. There are no additional risks to pregnant women or fetuses who are a part of this study. If you should become pregnant while already enrolled in the study, then you will be advised to discuss continuation in the study with your obstetrician.

Although efforts are made to protect your research study records, there is always a small risk that someone could get access to the personal information in your medical records or other information researchers have stored about you.

6. WHAT ARE THE POSSIBLE BENEFITS FROM THIS RESEARCH?

The possible benefit to you as a research participant in this research study is potentially being notified that you may have been recently infected with or exposed to SARS-CoV-2 during a time when you had few or no symptoms. Knowledge learned from this study may also be used to improve strategies to control this pandemic. However, there is no guarantee that you will benefit from being in this research.

7. WHAT ARE THE ALTERNATIVES TO TAKING PART IN THIS RESEARCH?

This research study is not designed to treat any medical condition that you may have. Therefore, there are no alternate procedure(s) or treatment(s) that would be advantageous to you. Your alternative is not to participate in this research.

8. IS THERE COMPENSATION FOR YOUR PARTICIPATION IN THIS RESEARCH?

Yes, for your participation, you will receive up to $55 per month: $25 upon completion of each clinic visit blood draw in the form of a gift card, and $10 for each of three completed online questionnaires (maximum of additional $30 per month for two symptom questionnaires and one risk exposure/PPE/social distancing questionnaire).

After August of 2021, at which point symptom questionnaires will have stopped, you will continue to be compensated up to $55 per visit. Compensation will be $45 per clinic visit and $10 for completing the risk exposure/PPE/social distancing questionnaire at least once since the last visit.

If the study team or doctor requests that you return to the clinic for an added appointment (in addition to your regular monthly clinic visit) this is considered an “unscheduled visit”. Unscheduled visits are only scheduled if needed and are compensated at $15 per unscheduled visit.

Unscheduled visit procedures may include *any of the evaluations and study procedures that would have taken place during a regular scheduled visit*, such as:

- Review of medical history and concomitant medications.
- Vital signs (temperature, pulse rate, and blood pressure) may be updated/collected.
- Obtain blood samples for research that we were unable to obtain at a prior scheduled visit.
- Adverse events may be documented.

Under 24 USC 30, payment to Federal Employees and Active Duty military personnel for participation in research while on duty is limited to blood donation and may not exceed $50 per blood draw. They may not receive any other payment or non-monetary compensation for participation in a research study unless they are off duty or on leave during the time they are participating in the protocol. However, if you are a recruit in training, you cannot be compensated, due to DoD policy, while during training. There are no restrictions on payment while off-duty. Active service members and Federal employees must complete the surveys during off-duty time in order to receive $10 compensation for questionnaires.

In order to receive payment for your participation in this study, you will be asked to complete a W-9 form. The W-9 form asks for your social security number and home address. The W-9 form will be stored in a locked cabinet at the study site and the Henry M. Jackson Foundation offices.

9. ARE THERE COSTS FOR PARTICIPATING IN THIS RESEARCH?

No, there are no costs to you for taking part in this research study.

10. WHAT WILL HAPPEN TO THE SAMPLES?

You are in a study where blood and saliva will be collected and stored for research. Blood samples will be separated into serum, plasma, and peripheral blood mononuclear cells (white blood cells), and cryopreserved. If you do NOT wish samples to be stored for future analyses related to COVID- 19, do not participate in this research study. By signing this consent document, you are also authorizing the storage of your biological specimens for future use in research studies related to COVID-19.

If samples are obtained, we will use the blood samples to look at how the body fights COVID-19 These tests will be conducted at a protocol-approved research laboratory, following applicable enhanced precaution standard operating procedures (SOPs). In addition to having your serum tested for antibodies using a research test at the Uniformed Services University, research studies will also be conducted to assess other aspects of your immune system, including evaluation of the numbers and functionality of immune cells such as T- cells and NK cells. We may also send your blood to commercial labs and/or academic institutions. This would be done to validate our antibody assay and other immunological studies, and to enable combination of the data obtained in this study with those of other COVID studies being conducted around the country. All specimens will be handled, stored, and disposed of in accordance with federal regulations.

Any data or specimen sent to outside commercial labs and/or academic institutions will be sent without identifiers so that no possible re-identification of these data or samples could be done by those institutions.

The storage (bank) area will be maintained at a protocol-approved biorepository or laboratory with an appropriate biosafety level and enhanced precaution SOPs. Other research investigators requesting samples from the bank must have a research protocol approved by their Institutional Review Board (IRB) (a committee responsible for protecting research participants).

We may also use the blood sample to analyze your DNA and other biological material to see how your immune system is able to fight COVID-19. We may compare your DNA with DNA from many other people in an attempt to determine what makes some people more susceptible to infection.

Since storage (banking) of biologic specimens for future genetic testing is still undergoing development, the risks of genetic testing are unknown. It is believed that the risks are very low. Using new technology, information about your DNA structure (genetic information) can be used to indicate risk for developing certain diseases. This genetic information is unique to you and may indicate changes in your future health status or life expectancy, or that of your children and other relatives. These discoveries could be stressful and cause psychological difficulties or family problems. It is also possible that during this research, people of your ethnic background may be found to be at more risk for certain diseases. Release of this information could stigmatize your ethnic or cultural group and may pose a possible risk of discrimination or increased difficulty in obtaining health insurance, life insurance, or employment. These risks would occur if the confidentiality of data is breached. *A Federal law, called the Genetic Information Nondiscrimination Act (GINA), generally makes it illegal for health insurance companies, group health plans, and most employers to discriminate against you based on your genetic information. Be aware that this law does not protect you against genetic discrimination by companies*

that sell life insurance, disability insurance, or long-term care insurance. Every effort will be made to protect the confidentiality of your medical information.

Generally, you will not be provided with the results of the studies using your stored samples. Any results from stored specimens would be of unclear value and unknown clinical meaning.

<u>Unspecified Future Research (future analyses not related to COVID-19)</u>
With your permission, we would also like to store your samples and use them for future ethically-approved medical research. The specifics of these research studies are unknown at this time, but these studies will frequently be in the area of new infectious diseases. Your samples will be stored with a study code number and cannot be traced back to you as the donor. The health information that would be stored include: your baseline medical history as documented on the medical intake form, whether or not you develop positive antibodies during the course of the study, and responses on the symptom and risk exposure/PPE/social distancing questionnaires. Your health information and biological samples will not be used for **unspecified future research** studies unless you give your permission by **initialing** your choices below.

<u>Health Information (**initial** preference below):</u>
______ I give permission to use my **health information** for unspecified future research
______ I DO NOT give permission to use my **health information** for unspecified future research

<u>Samples (**initial** preference below):</u>
______ I give permission to use my **samples** for unspecified future research
______ I DO NOT give permission to use my **samples** for unspecified future research

11. <u>CONFIDENTIALITY OF RECORDS OF STUDY PARTICIPATION:</u>

All information about you will be handled in confidence and only the people responsible for your care, specimen processing, and for this study will know that you were a part of the study. We will review your medical records and keep limited information about you in a secure file. All research information and samples will be labeled only with a number (study code) so that they cannot be directly linked to you personally.

Records of your participation in this study may only be disclosed in accordance with federal law, including the Federal Privacy Act, 5 U.S.C.552a, and its implementing regulations. DD Form 2005, Privacy Act Statement - Military Health Records, contains the Privacy Act Statement for the records.

By signing this consent document, you give your permission for information gained from your participation in this study to be published in medical literature, discussed for educational purposes, and used generally to further medical science. You will not be personally identified; all information will be presented as anonymous data.

Your records may be reviewed by your hospital, Uniformed Services University of the Health Sciences (USUHS) Institutional Review Board (IRB), Infectious Disease Clinical Research Program (IDCRP), authorized members of the Henry M. Jackson Foundation and their representatives, as well as other DoD and Federal regulatory agencies eligible to review research records as part of their responsibility to protect human participants in research.

We may report your results and samples to the Armed Forces Health Surveillance Branch, and the Centers for Disease Control and Prevention. The data and samples collected during this study may be looked at by state and local public health agencies.

Complete confidentiality cannot be promised, particularly for military personnel, because information regarding your health may be required to be reported to appropriate medical or command authorities, but every effort will be made to keep clinical records, research records, and other personal information confidential.

12. VOLUNTARY PARTICIPATION

The decision to take part in this research study is completely voluntary on your part which means you do not have to take part if you do not want to. You may also leave the research study at any time. If you choose not to take part in this research study or if you leave the study before it is finished, there will be no penalty or loss of benefits to which you are otherwise entitled.
You will be informed if significant new findings develop during the course of this research study that may relate to your decision to continue participation.

Can I request that I be withdrawn from the study at any point?

Yes, you can withdraw at any time. If you withdraw, the study team may reach out to ask you for the reason for withdrawal. You can state you do not want to give a reason or simply not respond.

Samples and data collected before your withdrawal will be kept for research.

The study Principal Investigator may also terminate your participation in this study at any time if he/she feels this to be in your best interest or due to the military mission.

****

13. AUTHORIZATION TO USE AND/OR DISCLOSE PROTECTED HEALTH INFORMATIONFORRESEARCH(HIPAA):

Principal Investigator (PI) Name and Rank: [redacted]
Corps and Service/Department: Uniformed Services University (USUHS)
Title of Research Project: *Prospective Assessment of SARS-CoV-2 Seroconversion (PASS)*

I. Purpose of this Document

An Authorization is your signed permission to use or disclose your health information. The Health Insurance Portability and Accountability Act (HIPAA) Privacy Rule, as implemented by the Department of Defense (DoD), permits the Military Health System (MHS) to use or disclose your health information with a valid Authorization. The MHS is defined as all DoD health plans and DoD health care providers that are organized under the management authority of, or in the case of covered individual providers, assigned to or employed by, the Defense Health Agency (DHA), the Army, the Navy, or the Air Force. A valid Authorization must include the core elements and required statements as contained in this document.

Please read the information below and ask questions about anything you do not understand before deciding to give permission for the use and disclosure of your health information.

II. Authorization

The following describes the purposes of the requested use and disclosure of your health information:

The goal of this study is to better understand how common SARS-CoV-2 infection is among healthcare workers, clinical presentation, and immune responses in healthcare workers at the Walter Reed National Military Medical Center (WRNMMC) over the course of one year.

A. What health information will be used or disclosed (released) about you?

• Names	• Health plan beneficiary numbers
• Address	• Phone numbers
• Date of birth	• Fax numbers
• Age	• E-mail addresses
• DoD identification number	• Last 4 digits of social security number
• Health information	• Medical record numbers

B. Who will be authorized to use or disclose (release) your health information?

- MTFs who have treated you in the past

C. Who may receive your health information?

- The Institutional Review Board (USUHS IRB)
- Infectious Disease Clinical Research Program (IDCRP)
- Other Military Treatment Facilities or Department of Defense representatives
- Henry M. Jackson Foundation and their representatives
- Local, State and Federal Government representatives, when required by law
- Defense Health Agency (DHA)
- Food and Drug Administration (FDA)
- Centers for Disease Control and Prevention (CDC)
- National Institute of Allergy and Infectious Disease (NIAID) and their representatives
- Naval Medical Research Center Clinical Trial Center (NMRC CTC)
- Walter Reed National Military Medical Center (WRNMMC)

D. What if you decide not to sign this authorization?

- You do not have to sign this Authorization, but if you do not, you will not be allowed to participate in this research study because your health information is required for your participation.
- The MHS **will not** condition (withhold or refuse) treatment that is not part of this study, payment, enrollment, or eligibility for benefits on whether you sign this Authorization.

E. Is your health information requested for future research studies?

- Yes, your health information ***is*** requested for use or disclosure (release) in future research studies as specified below:

i. Qualified investigators who want to investigate future analyses related to COVID-19.

Your health information will not be used for future research studies unless you give your permission by **initialing** your choice below:

____________________I give permission to use my health information for future research studies

____________________I ***do not*** give permission to use my health information for future research studies

F. Can you access your health information during the study?

You may have access to your health information at any time unless your identifiers are permanently removed from the data.

G. Can you revoke this authorization?

- You may change your mind and revoke (take back) your Authorization at any time except to the extent that the MHS has already acted in reliance on your Authorization. Even if you revoke this Authorization, any person listed above who received your Authorization for purposes of the research study may still use or disclose (release) any already obtained health information as necessary to maintain the integrity or reliability of this research.
- If you revoke this Authorization, you may no longer be allowed to participate in this research study.
- If you want to revoke your Authorization, you must write to:
  i. The Study Doctor who is the Principal Investigator at your medical site (name and complete mailing address in the Contact section of this form) to inform him/her of your decision.

H. Does this authorization expire?

- No, it does not expire.

I. What else may you want to consider?

- No publication or public presentation about the research described above will reveal your identity without another signed Authorization from you.
- If all information that does or can identify you is removed from your health information, the remaining de-identified information will no longer be subject to this Authorization and may be used or disclosed (released) for other purposes.
- Once your health information is shared or disclosed (released) outside of the MHS, the privacy of your health information cannot be guaranteed, and it may no longer be protected by the Federal privacy laws (such as the HIPAA Privacy Rule).

Signature of Research Participant or Personal Representative:

Your signature acknowledges that:

- You authorize the MHS to use and disclose (release) your health information for the research purposes stated above.
- You have read (or someone has read to you) the information in this Authorization.
- You have been given a chance to ask questions, and all of your questions have been answered to your satisfaction.

______________________________ _______________

Participant Signature Date

If the personal representative signs on a participant's behalf, then the personal representative must provide verification of their authority under applicable state law.

______________________________ _______________

Personal Representative Signature Date

______________________________ ______________________________

Personal Representative Printed Name Description of the personal representative's authority

If you have not already received a copy of the brochure entitled "Military Health System Notice of Privacy Practices," you may request one. DD Form 2005, Privacy Act Statement - Military Health Records (located on your medical records jacket), contains the Privacy Act Statement for the records. If you have any questions or concerns about your privacy rights, you should contact:

Uniformed Services University Human Research Protections Program
NMRC Human Research Protections Officer [redacted]

14. WHAT HAPPENS IF YOU ARE INJURED AS A RESULT OF THIS RESEARCH?

If you think that you have a research-related injury, notify your Principal Investigator immediately using the contact information in the section below.

If you are injured because of your participation in this research and you are a DoD healthcare beneficiary (e.g., active duty military, dependent of active duty military, retiree), you are authorized space-available medical care for your injury within the DoD healthcare system, as long as you remain a DoD healthcare beneficiary. This care includes, but is not limited to, free medical care at DoD hospitals or DoD clinics.

If you are injured because of your participation in this research and you are not a DoD healthcare beneficiary, you are authorized space-available medical care for your injury at a DoD hospital or a DoD clinic; medical care charges for care at a DoD hospital or a DoD clinic will be waived for your research-related injury. If you obtain care for research-related injuries outside of a DoD or DoD hospital or clinic, you will not be reimbursed for those medical expenses.

For DoD healthcare beneficiaries and non-DoD healthcare beneficiaries: Transportation to and from hospitals or clinics will not be provided or paid for by the DoD or IDCRP. Unless you are covered by TRICARE, no DoD reimbursement is available if you incur medical expenses to treat research-related injuries. No compensation is available for research-related injuries. You are not waiving any legal rights.

15. CONTACT INFORMATION:

Principal Investigator (PI)

The Principal Investigator or a member of the research staff will be available to answer any questions throughout this study.

Principal Investigator: [redacted]
Phone: [redacted]
Mailing Address: [redacted]

NMRC Human Research Protection Program (HRPP) Office

The Human Research Protection Program Office Point of Contact and/or Human Protections Administrator (HPA) will be available to answer questions or discuss concerns you may have about this research study.

Human Protections Administrator/HRPP POC: [redacted]
Phone: [redacted]

Institutional Review Board (IRB) Office

If you have any questions about your rights as a research participant or if you have concerns or complaints about the research study, please contact the USUHS Institutional Review Board Office at: [redacted]

(USUHS) IRB 1
IRB NUMBER: IDCRP-126
IRB APPROVAL DATE: 01/12/2021

IF THERE IS ANY PORTION OF THIS DOCUMENT THAT YOU DO NOT UNDERSTAND, ASK THE INVESTIGATOR BEFORE SIGNING. YOU MAY CONSULT WITH YOUR PERSONAL PHYSICIAN OR LEGAL ADVISOR, IF YOU WISH.

A signed and dated copy of this document will be given to you.

SIGNATURE OF PARTICIPANT

By signing below, I agree that I have been provided time to read the information describing the research study in the consent form. The content and meaning of this information has been explained to me. I have been provided with the opportunity to ask questions. I voluntarily consent to participate in this study.

By signing this form, I have not given up any of my legal rights as a research participant.

____
Printed Name of Participant

____ _______________
Signature of Participant Date

Your signature below indicates you are legally authorized to act on behalf of the participant and have read this document. You will receive a copy of this document.

SIGNATURE OF INDIVIDUAL ADMINISTERING CONSENT
(Can only be signed by an investigator or staff approved to administer consent)

____
Printed Name of Administering Individual

____ __________________________
Signature of Administering Individual Date Time (hh:mm)

(USUHS) IRB 1
IRB NUMBER: IDCRP-126
IRB APPROVAL DATE: 01/12/2021
