# Supplementary material for: Prospective Assessment of SARS-CoV-2 Seroconversion (PASS) study: an observational cohort study of SARS-CoV-2 infection and vaccination in healthcare workers
Source: BMC Infect Dis. 2021 Jun 9;21:544. doi: 10.1186/s12879-021-06233-1 (PMC8188741; doi:10.1186/s12879-021-06233-1)
Supplement: Supplementary file 3 — Additional file 3. Informed consent addendum. [file 12879_2021_6233_MOESM3_ESM.pdf]

*Naval Medical Research Center Clinical Trial Center (NMRC CTC)*
**Title:** *Prospective Assessment of SARS-CoV-2 Seroconversion (PASS)*
**CONSENT ADDENDUM – MAIN CONSENT FORM SUPPLEMENT**
**Principal Investigator:** [redacted]

Please take time to carefully review this information. You should talk to the researchers about the research study and ask them any questions you have. You may also wish to talk to others (for example, your friends, family, or your personal physician) about your potential participation in this research study. You do not have to take part in this study. Participation is voluntary. You may also leave the research study at any time without penalization.

## 1. <u>KEY INFORMATION:</u>

Thank you for being a research participant in the Prospective Assessment of SARS-CoV-2 Seroconversion study (PASS). The goal of the PASS study is to better understand the frequency, clinical presentations, and immune responses of SARS-CoV-2 infection in healthcare staff at Walter Reed National Military Medical Center (WRNMMC).

*As many PASS study subjects are now getting vaccinated against COVID-19, we now want to also determine if previous exposure to seasonal coronaviruses or other aspects of baseline immune function affects the strength and duration of vaccine-induced immune responses. We will also be able to determine for how long people have detectable antibody responses to SARS-CoV-2 after vaccination.*

### *PURPOSE OF THIS ADDENDUM*

You are being asked to sign this consent addendum because you are participating in a research study at WRNMMC entitled “PASS”. This consent addendum is a supplement to the main informed consent form. This consent addendum invites you to continue to take part in the PASS study for up to a total of four years (potentially up to August of 2024), with monthly visits until August of 2021, and then more spaced out visits (quarterly for year two and semiannually for years 3 and 4).

- ❖ Signing this consent addendum does not obligate you to continue participating in the PASS study for this duration of time. You may withdraw at any time.
- ❖ To enable us to compare immune responses in individuals that become infected, vaccinated, or neither, we are now going to follow all subjects on the same schedule.
- ❖ In this consent addendum we are also asking whether you would like to be invited to virtual update meetings for the PASS study and/or receive PASS study updates by email.



**(USUHS) IRB 1**
**IRB NUMBER: IDCRP-126**
**IRB APPROVAL DATE: 01/12/2021**

Taking part in this research is entirely voluntary. If you choose not to participate in this research study, your decision will not affect your eligibility for care or any other benefits to which you are entitled. Please read the information below, and ask questions about anything you do not understand, before deciding whether or not to take part in the study.

**The primary question this <u>additional</u> part of the study hopes to answer is:**

1) Do pre-existing immune responses from exposure to seasonal coronaviruses affect the strength or duration of antibody responses from a COVID-19 vaccine?

**Other questions this <u>additional</u> part of the study may be able to answer include:**

1) For how long do COVID-19 vaccines induce detectable antibody and T-cell responses against SARS-CoV-2?
2) Are there baseline immunological factors that can be used to predict local and systemic reactions to COVID-19 vaccines?
3) Are there baseline immunological factors or immune responses that develop soon after COVID-19 vaccination that can be used to predict the strength of vaccine-induced antibody responses?
4) Does prior infection with SARS-CoV-2 change the immune responses to COVID-19 vaccine?
5) Are there differences in the strength and duration of the immune responses caused by different COVID-19 vaccines?
6) What is the effectiveness of COVID-19 vaccines in protecting against COVID-19 disease?

If you decide to take part in this part of the research study, you will be asked to sign this consent addendum. Before you sign this document, be sure you understand what the study addendum is about in all sections of the consent form, including the risks and possible benefits to you.

## 2. <u>WHAT IS THE PURPOSE AND DURATION OF THIS RESEARCH AND WHO WILL TAKE PART?</u>

You are being asked to take part in this research study because you are currently enrolled as a study subject in the PASS study.

We are asking for your consent to continue to be followed in this study now that healthcare personnel are being vaccinated against COVID-19 so that we can study immune responses to COVID-19 vaccines. Because we are interested in learning about how long vaccine-induced immune responses last, we are obtaining consent to potentially follow study subjects for as long as four years. Signing this consent does not obligate you to remain in this study for that time period – you may withdraw at any point. *Also, please be aware that the actual duration of the study will be dependent on funding availability.*

For study subjects, the duration of participation per visit will be about one hour per session for one visit a month until August of 2021. In year two, you will have about four visits, and in years 3 and 4 of the study you will have two visits per year.

You will be asked at every clinic visit to share the results of any COVID-19 tests that have been conducted on yourself outside this research study since the last clinic visit. You will also be asked about any COVID-19 vaccines, other medications, medical issues, or other vaccines you may have gotten since your last visit.

The research results about you will not be shared with you. Please be aware that COVID-19 vaccines are new and that at this time we do not yet know what immune responses after vaccination are predictive of protection against COVID-19 disease. For example, we do not know if development of anti-SARS-CoV-2 antibodies detectable with our assay are associated with protection against COVID-19 disease. Similarly, we do not know if a drop-off in previously detectable post-vaccination antibody levels correlates with increased susceptibility to COVID-19. As such, after being COVID-19 vaccinated you will no longer be informed of the results of your antibody responses to SARS-CoV-2. If in the future we learn that poor antibody responses after vaccination are an indication for repeat vaccination, then we will amend our protocol and inform you of your antibody results at that time. Published papers based on the results of this study may be shared with study subjects via PASS study email newsletters.

## 3. SCREENING PROCESS TO QUALIFY FOR CONTINUED PARTICIPATION IN THIS STUDY

All PASS participants who elect to continue in the study after they receive a COVID-19 vaccine are eligible to do so.

## 4. WHAT WILL HAPPEN IF YOU DECIDE TO CONTINUE TO BE IN THIS RESEARCH STUDY?

Your medical records may be reviewed now and in the future for information related to COVID- 19 and concurrent and past conditions and medications. We will ask you about your health and will collect blood and saliva for research testing purposes.

For all subjects who continue to be followed in the PASS study after COVID-19 vaccination:

Revised study schedule details
(1) All study subjects, regardless of infection or vaccination status, will continue to be followed once a month until August, 2021. At that point, all subjects will change to being seen quarterly (about once every three months) in the clinic for the 2nd year of the study. Subjects remaining in the study during years 3 and 4 will be asked to return to the clinic just twice a year during those years.

Subjects that are vaccinated against COVID-19 or diagnosed with SARS-CoV-2 infection from May to August of 2021 may be asked to continue monthly clinic visits for an additional three months before switching to quarterly visits.

Until August of 2021, we ask that study participants continue the twice a month (and when symptomatic) symptom questionnaires, the once a month risk exposure/PPE/social distancing questionnaire, and return to clinic monthly when possible.

***Please note that the investigators understand that it can be challenging to return to the study clinic every month. Study subjects are welcome to continue to be followed in the study even if they are unable to make it to all monthly visits.***



**(USUHS) IRB 1**
**IRB NUMBER: IDCRP-126**
**IRB APPROVAL DATE: 01/12/2021**

(2) Study subjects that receive a COVID-19 vaccine will be asked to fill out a COVID-19 vaccine questionnaire after each vaccination.

(3) After August of 2021, symptom questionnaires will stop.

(4) After August of 2021, we will ask you to complete a risk exposure/PPE use/social distancing questionnaire once every three months during year 2, and then twice a year for years 3 and 4.

(5) Study subjects are asked to continue to report to the WRNMMC COVID-19 testing center whenever experiencing symptoms consistent with COVID-19, even if they have already been COVID-19 vaccinated, for as long as the facility is available.

At each study visit you will complete the following study procedures:

- Discuss with the clinic team any issues with filling out the on-line questionnaires, notify the team if you have been tested for COVID-19 since the last visit, notify the team if you have been vaccinated against COVID-19, fill out a COVID-19 vaccination form if you have been vaccinated, and update the team with regards to any major changes to your health or medication use since the last visit
- Possibly provide saliva for SARS-CoV-2 antibody testing
- Provide blood samples for SARS-CoV-2 antibody testing and other immunological tests

Revised timeline of visits and samples to be collected:

Schedule of Visits and Samples to be Obtained

- All study subjects will be followed in the clinic once a month through August 2021, and then switched to more spaced out visits
- Symptom questionnaires will end in August 2021
- Risk exposure questionnaires will continue quarterly or semiannually after August 2021

| | | **Year 1** | **Year 2** | **Years 3 and 4** |
|---|---|---|---|---|
| **Clinic Visit Schedule** | **Baseline Visit** | **Every month through August 2021** | **Every three months through August 2022** | **Every six months through August 2024** |
| **Risk Exposure Questionnaire** | Monthly | Monthly | Quarterly* | Semiannually |
| **Symptoms Review Questionnaire** | Yes and daily if symptoms present | Yes and daily if symptoms present | No | No |
| **Saliva for Coronavirus IgA** | Yes (one tube) | 0-1 tubes | 0-1 tubes | 0-1 tubes |
| **Serum Separator Tubes (SST) for COVID multiplex serology analysis** | 1 x 7.5ml | 1 x 7.5ml | 1 x 7.5ml | 1 x 7.5ml |
| **Mononuclear Cell Preparation Tube (CPT Na Citrate or CPT Na Heparin) for PBMCs and plasma isolation** | 4-6 x 8ml | 0-6 x 8ml | 0-6 x 8ml | 0-6 x 8ml |
| **PAXgene for WB RNA transcriptomics** | 1 x 3ml | 0-1 x 3ml | 0-1 x 3ml | 0-1 x 3ml |
| **WB in EDTA tube for DNA analysis of B cell and T cell repertoires** | 1 x 2ml | 0-1 x 2ml | 0-1 x 2ml | 0-1 x 2ml |
| **Total blood volume (ml)** | 44.5ml - 60.5ml | 7.5ml - 60.5ml | 7.5ml - 60.5ml | 7.5ml - 60.5ml |

* Subjects that become COVID-19 infected or vaccinated after April of 2021 may be asked to continue monthly visits for the first quarter of year 2



**(USUHS) IRB 1**
**IRB NUMBER: IDCRP-126**
**IRB APPROVAL DATE: 01/12/2021**

Boxes in white --> samples we expect to always obtain at each of these timepoints
Boxes in grey --> what we expect to obtain intermittently (especially at key timepoints after vaccination or infection)

**Details on when we anticipate drawing a full set of saliva and blood samples (grey boxes)**

- For individuals that remain uninfected and unvaccinated --> once yearly draw all blood and saliva tubes except for the PAXgene tube
- For individuals that test COVID-19 positive by PCR, antigen, or antibody test
  - At first monthly visit after infection draw all blood and saliva tubes, including both the EDTA and PAXgene tubes.
  - At visits at 3 and 6 months after positive test, twice yearly in year 2, and yearly in years 3 and 4 draw all blood and saliva tubes except the PAXgene tube.
- For all individuals vaccinated against COVID 19
  - At visits 1, 6, and 12 months after final vaccination, twice yearly in year 2, and yearly in years 3 and 4 draw all blood and saliva tubes except the PAXgene tube
- Individuals that come to clinic within one week of their first COVID-19 vaccination draw a full set of blood and saliva tubes, including both the PAXgene and EDTA tubes
- Individuals that come to clinic within one week of their second COVID-19 vaccination draw a saliva tube, an SST tube, a PAXGene tube, and an EDTA tube

*Note: The exact tubes to be drawn at each follow-up visit may change as determined by the primary investigator, but will not exceed 60.5 ml.*

## 5. IS THERE COMPENSATION FOR YOUR CONTINUED PARTICIPATION IN THIS RESEARCH STUDY?

Yes, for your participation, you will receive up to $55 per clinic visit: Until August of 2021, you will receive $25 upon completion of each clinic visit blood draw in the form of a gift card, and $10 for each of three completed online questionnaires (maximum of additional $30 per visit for two symptom questionnaires and one risk exposure/PPE/social distancing questionnaire).

*After August of 2021, at which point symptom questionnaires will have stopped, you will continue to be compensated up to $55 per visit. Compensation will be $45 per clinic visit and $10 for completing the risk exposure/PPE/social distancing questionnaire at least once since the last visit.*

If the study team or doctor requests that you return to the clinic for an added appointment (in addition to your regular monthly clinic visit) this is considered an "unscheduled visit". Unscheduled visits are only scheduled if needed and are compensated at $15 per unscheduled visit.

Unscheduled visit procedures may include *any of the evaluations and study procedures that would have taken place during a regular scheduled visit,* such as:

- Review of medical history and concomitant medications.
- Vital signs (temperature, pulse rate, and blood pressure) may be updated/collected.
- Obtain blood samples for research that we were unable to obtain at a prior scheduled visit.
- Adverse events may be documented.



**(USUHS) IRB 1**
**IRB NUMBER: IDCRP-126**
**IRB APPROVAL DATE: 01/12/2021**

Under 24 USC 30, payment to Federal Employees and Active Duty military personnel for participation in research while on duty is limited to blood donation and may not exceed $50 per blood draw. They may not receive any other payment or non-monetary compensation for participation in a research study unless they are off duty or on leave during the time they are participating in the protocol. However, if you are a recruit in training, you cannot be compensated, due to DoD policy, while during training. There are no restrictions on payment while off-duty. Active service members and Federal employees must complete the surveys during off-duty time in order to receive $10 compensation for questionnaires.

In order to receive payment for your participation in this study, you will be asked to complete a W-9 form. The W-9 form asks for your social security number and home address. The W-9 form will be stored in a locked cabinet at the study site and the Henry M. Jackson Foundation offices.

## 6. ELECTION TO BE INVITED TO VIRTUAL PASS STUDY UPDATE MEETINGS AND/OR TO RECEIVE PASS STUDY UPDATES BY EMAIL

The PASS investigators are considering having virtual update meetings and/or electronic email newsletters for PASS study participants. We anticipate these meetings and/or newsletters would be held and/or distributed about once every 3-6 months. Attendance at virtual meetings would be completely voluntary. The meetings and/or newsletters would enable PASS subjects to hear about the ongoing progress of the study, including:

- The goals of the study
- Details of the protocol design, including any changes to the protocol
- Enrollment and retention numbers to date
- Presentation and discussion of any published study results

Virtual meetings would also include time for participants to ask questions if they need clarification on any of the study protocols.

If you are interested in attending virtual meetings for PASS study participants, please know that <u>attendance at virtual meetings will result in your participation in the study being identifiable by others</u>.

Additionally, while we will do our best to send email newsletters such that your email address is not seen by others, there is a risk that distribution of email newsletters results in your email being visible to others in the study.

**For possible PASS study virtual update meetings and email newsletters, please elect one of the following:**

**___________ (initials) I <u>consent to receiving</u> newsletters and invitations to virtual PASS study meetings by email, and understand that attendance at virtual meetings would result in my participation in this study being publicly known by others.**

**___________ (initials) I elect <u>not</u> to receive PASS study newsletters or invitations to virtual PASS study meetings.**



**(USUHS) IRB 1**
**IRB NUMBER: IDCRP-126**
**IRB APPROVAL DATE: 01/12/2021**

## 7. VOLUNTARY PARTICIPATION

The decision to continue to take part in this research study is completely voluntary on your part which means you do not have to take part if you do not want to. You may also leave the research study at any time. If you choose not to take part in this research study or if you leave the study before it is finished, there will be no penalty or loss of benefits to which you are otherwise entitled.
You will be informed if significant new findings develop during the course of this research study that may relate to your decision to continue participation.

Can I request that I be withdrawn from the study at any point?

Yes, you can withdraw at any time. If you withdraw, the study team may reach out to ask you for the reason for withdrawal. You can state you do not want to give a reason or simply not respond.

Samples and data collected before your withdrawal will be kept for research.

The study Principal Investigator may also terminate your participation in this study at any time if he/she feels this to be in your best interest or due to the military mission.

## 8. CONTACT INFORMATION:

### Principal Investigator (PI)

The Principal Investigator or a member of the research staff will be available to answer any questions throughout this study.

*Principal Investigator:* [redacted]
*Phone:* [redacted]
*Email:* [redacted]
*Mailing Address:* [redacted]

### NMRC Human Research Protection Program (HRPP) Office

The Human Research Protection Program Office Point of Contact and/or Human Protections Administrator (HPA) will be available to answer questions or discuss concerns you may have about this research study.

*Human Protections Administrator/HRPP POC:* [redacted]

### Institutional Review Board (IRB) Office

If you have any questions about your rights as a research participant or if you have concerns or complaints about the research study, please contact the USUHS Institutional Review Board Office at: [redacted]

IF THERE IS ANY PORTION OF THIS CONSENT ADDENDUM THAT YOU DO NOT UNDERSTAND, ASK THE INVESTIGATOR BEFORE SIGNING. YOU MAY CONSULT WITH YOUR PERSONAL PHYSICIAN OR LEGAL ADVISOR, IF YOU WISH.
A signed and dated copy of this consent addendum will be given to you.

**SIGNATURE OF PARTICIPANT**

By signing below, I agree that I have been provided time to read the information describing the questions this additional part of the study hopes to answer. The content and meaning of this information has been explained to me. I have been provided with the opportunity to ask questions. I voluntarily consent to participate in this additional part of the study.

By signing this consent addendum, I have not given up any of my legal rights as a research participant.

________________________________________
Printed Name of Participant

________________________________________ ______________
Signature of Participant Date

Your signature below indicates you are legally authorized to act on behalf of the participant and have read this document. You will receive a copy of this consent addendum.

**SIGNATURE OF INDIVIDUAL ADMINISTERING CONSENT**
(Can only be signed by an investigator or staff approved to administer consent)

________________________________________
Printed Name of Administering Individual

________________________________________ ____________________________
Signature of Administering Individual Date Time (hh:mm)



**(USUHS) IRB 1**
**IRB NUMBER: IDCRP-126**
**IRB APPROVAL DATE: 01/12/2021**
