# Supplementary material for: Prospective Assessment of SARS-CoV-2 Seroconversion (PASS) study: an observational cohort study of SARS-CoV-2 infection and vaccination in healthcare workers
Source: BMC Infect Dis. 2021 Jun 9;21:544. doi: 10.1186/s12879-021-06233-1 (PMC8188741; doi:10.1186/s12879-021-06233-1)
Supplement: Supplementary file 4 — Additional file 4. Medical intake form. [file 12879_2021_6233_MOESM4_ESM.pdf]

SUBJECT ID#: P126-S00- ☐☐☐☐

This form is completed via interview with the subject at SCREENING and reviewed at BASELINE.

Demographics

Age: ________

Sex: ☐Male ☐Female

Birth date: __ __ / __ __ __ / __ __ __ __ (dd/MMM/yyyy)

Ethnicity: ☐Hispanic or Latino ☐Not reported ☐Not Hispanic or Latino ☐Unknown

Indicate No or Yes for each race listed below. Yes may be checked for more than one race. If a subject refuses to identify his or her race, check No to all options.

American Indian or Alaskan Native: ☐Yes ☐No

Asian: ☐Yes ☐No

Native Hawaiian or other Pacific Islander: ☐Yes ☐No

Black or African American: ☐Yes ☐No

White: ☐Yes ☐No

Occupation: ____

Vaccination History & Other COVID-19 Studies Enrollment Status

Have you received any vaccines in the past month? ☐*Yes ☐No

If *Yes, list vaccines:____

____

COVID-19 vaccine or therapy trial enrollment status:

☐Previously enrolled

☐Currently enrolled

☐Plan to enroll soon (i.e. in the next one or two months)

☐N/A

CRF ENTRY	CRF QC	DB ENTRY	DB QC
Initial/date	Initial/date	Initial/date	Initial/date

SUBJECT ID#: P126-S00- ☐☐☐☐

**Medical History - Comorbidities & Risk Factors**					
**Asthma**	☐*Yes ☐No				
**If *Yes:**	☐Well-controlled (defined as <2 days per week etc.) ☐Not well controlled				
**Pulmonary Disease**	*Any other pulmonary disease (including emphysema, chronic bronchitis, and interstitial lung disease).*<br>☐Yes ☐No ☐Ongoing ☐Unknown				
**Diabetes**	☐*Yes ☐No				
**If *Yes:**					
Insulin Requiring	☐Yes ☐No				
Controlled	☐Well-controlled (HgbA1c<7% in the last 6 months) ☐Not well controlled				
**Kidney Disease**	☐*Yes ☐No				
**If *Yes:**					
Severity	☐Mild (GFR <60) ☐Greater than mild				
**Liver Disease**	*Any liver disease, including fatty liver disease, cirrhosis, or fibrosis.*<br>☐Yes ☐No ☐Ongoing ☐Unknown				
**Cancer**	*Any type of cancer that is not in complete remission (e.g. excision of basal cell carcinoma, breast mass, benign cancerous lesions) due to total excision of lesion or no evidence of cancer for at least 5 years.*<br>☐Yes ☐No ☐Ongoing ☐Unknown				
**Cardiovascular Disease**	Do you have coronary artery disease?	☐Yes	☐No	☐Ongoing	☐Unknown
	Have you ever had a heart attack?	☐Yes	☐No	☐Ongoing	☐Unknown
	Do you have angina?	☐Yes	☐No	☐Ongoing	☐Unknown
	Do you have valvular disease?	☐Yes	☐No	☐Ongoing	☐Unknown
	Do you have an abnormal heart rhythm?	☐Yes	☐No	☐Ongoing	☐Unknown
	Do you have a pacemaker?	☐Yes	☐No	☐Ongoing	☐Unknown
	Any other cardiac disease?	☐*Yes	☐No	☐Ongoing	☐Unknown
	**If *Yes,** ________________________________				

CRF ENTRY	CRF QC	DB ENTRY	DB QC
Initial/date	Initial/date	Initial/date	Initial/date

SUBJECT ID#: P126-S00- ☐☐☐☐

**Heart Failure**	☐*Yes ☐No
**If *Yes:**	
Severity	*Ever diagnosed with greater than or equal to Class 2 heart failure (mild symptoms and slight limitations during normal activity).*<br>☐Yes ☐No ☐Ongoing ☐Unknown
**Hypertension**	☐*Yes ☐No
**If *Yes:**	
Severity	*Ever diagnosed with Grade 2 (moderate) or 3 (severe) hypertension with blood pressure readings consistently greater than 160/100 mmHg even with the use of medications and lifestyle changes.*<br>☐Yes ☐No ☐Ongoing ☐Unknown
**Immunocompromised State or Immune Modulating Medications**	*Ever diagnosed with an immunocompromised state or use of immune modulating medications within the last 12 months.*<br>☐Yes ☐No ☐Ongoing ☐Unknown
**Smoking (ever)**	☐*Yes ☐No (never smoked)
**If *Yes:**	Average number of packs per day: ☐ Less than a pack a day **or more**, if more specify # of packs/day _____<br>Smoking, number of years: _____<br>Packs/Year history (average # packs per day times # of years smoked): _____<br>Currently smoking?<br>☐*Yes ☐*No<br>**If *No**, when did you stop: __ __ / __ __ __ / __ __ __ __ (dd/MMM/yyyy)
**Vaping (ever)**	☐*Yes ☐No
**If *Yes:**	When did you start: __ __ / __ __ __ / __ __ __ __ (dd/MMM/yyyy)
**Pregnancy**	Are you pregnant?<br>☐Yes ☐No ☐Unknown
**Children**	Do you have any children?<br>☐*Yes ☐No<br>**If *Yes**, how many: ______<br>**What is the age of each child living in your household?**<br>Child 1:____ Child 2:____ Child 3:____ Child 4:____ Child 5:____ Child 6:____ Child 7:____
**Number of People**	How many people are living in your household, including yourself? __________

CRF ENTRY	CRF QC	DB ENTRY	DB QC
Initial/date	Initial/date	Initial/date	Initial/date

SUBJECT ID#: P126-S00- ☐☐☐☐

Medical History - Comorbidities & Risk Factors - CONTINUED

**Anemia**	☐Yes ☐No	**High Cholesterol**	☐Yes ☐No
**Arthritis** ☐*Yes ☐No	**If Yes***, specify ☐Inflammatory ☐Non-inflammatory	**HIV/AIDS**	☐Yes ☐No
		**Kidney Stones**	☐Yes ☐No
**Clotting Disorder**	☐Yes ☐No	**Stroke**	☐Yes ☐No
**Epilepsy**	☐Yes ☐No	**Thyroid Disease**	☐Yes ☐No
**Other Neurological Disease**	☐*Yes ☐No<br>**If *Yes**, specify<br>____________________	**Autoimmune Disease**	☐*Yes ☐No<br>**If *Yes**, specify<br>____________________
**Do you have any environmental allergies?**	☐*Yes ☐No<br>**If *Yes**, to what:<br>____________________	**Do you have any food allergies?**	☐*Yes ☐No<br>**If *Yes**, to what:<br>____________________
**Do you have any medication allergies?**	☐*Yes ☐No<br>**If *Yes**, to what:<br>____________________		

Please list any past surgeries you had:

**1.**	**8.**
**2.**	**9.**
**3.**	**10.**
**4.**	**11.**
**5.**	**12.**
**6.**	**13.**
**7.**	**14.**

CRF ENTRY	CRF QC	DB ENTRY	DB QC
Initial/date	Initial/date	Initial/date	Initial/date

SUBJECT ID#: P126-S00- ☐☐☐☐

Presence of Any Current Symptoms

**Symptoms**	Symptoms of fever, cough, anorexia, myalgias, chills, shortness of breath, anosmia, sore throat, rhinorrhea, or diarrhea at enrollment	☐Yes ☐No	☐Ongoing ☐Unknown
**Fever**	Presence of fever (T > 100.4 °F) on screening vital signs	☐Yes ☐No	☐Ongoing ☐Unknown
**COVID-19 Diagnosis**	Known prior diagnosis with COVID-19	☐Yes ☐No	☐Ongoing ☐Unknown

Weight and Height Measurement

Was weight assessed? ☐Yes ☐No

If No, reason not done: ☐Subject unable to comply ☐ Investigator decision ☐Subject refusal ☐ Other, specify: ____________________

Date assessed: __ __ / __ __ __ / __ __ __ __ (dd/MMM/yyyy)

☐Pounds __ __ __ . __ **lb** OR

☐Kilograms __ __ __ . __ **kg**

Was height assessed? ☐Yes ☐No

☐Inches __ __ __ . __ **in** OR

☐Centimeters __ __ __ . __ **cm**

Body Mass Index (BMI)

BMI: ___ ___

Vital Signs

Respiratory Rate: ___ ___

O_2 Saturations: ___ ___

Any supplemental oxygen? ☐Yes ☐No

Temperature: ___ ___ . ___ ☐°C ☐°F

Heart Rate: ___ ___ ___

Diastolic BP: ___ ___ ___

Systolic BP: ___ ___ ___

CRF ENTRY	CRF QC	DB ENTRY	DB QC
Initial/date	Initial/date	Initial/date	Initial/date

SUBJECT ID#: P126-S00- ☐☐☐☐

CURRENT MEDICATIONS LIST

☐N/A

____

PAST MEDICAL HISTORY

☐N/A

____

ELIGIBILITY

Eligible to participate? ☐Yes ☐No

SEROLOGY RESULTS

IgG ☐Positive ☐Negative

IgM ☐Positive ☐Negative

COMMENTS

____

CRC Signature:______________________ Date:______________

CRC Signature:______________________ Date:______________

CRC Signature:______________________ Date:______________

CRF ENTRY	CRF QC	DB ENTRY	DB QC
Initial/date	Initial/date	Initial/date	Initial/date
