# Supplementary material for: Prospective Assessment of SARS-CoV-2 Seroconversion (PASS) study: an observational cohort study of SARS-CoV-2 infection and vaccination in healthcare workers
Source: BMC Infect Dis. 2021 Jun 9;21:544. doi: 10.1186/s12879-021-06233-1 (PMC8188741; doi:10.1186/s12879-021-06233-1)
Supplement: Supplementary file 5 — Additional file 5. Monthly CTC visit questionnaire. [file 12879_2021_6233_MOESM5_ESM.pdf]

PASS Study Monthly Clinic Visit Questions

SUBJECT ID#: P126-S00-☐☐☐☐ Visit Date: _ _ / _ _ _ / _ _ _ _ Visit #: ____

1. Since your last clinic visit, have you received a COVID-19 vaccination?

☐*Yes ☐No ***If *Yes, please fill out a COVID-19 vaccination history form.***

2. Since your last clinic visit, have you received any other vaccinations?

☐*Yes ☐No

If ***Yes**, please specify:

Vaccine Name	Vaccine Indication

3. Since your last clinic visit, have you enrolled in any other COVID-19 trial(s)?

☐*Yes ☐No ☐*No, but plan to enroll soon (i.e. in the next one or two months)

If ***Yes** or ***No, but plan to enroll soon**, does it involve experimental vaccination or medication for COVID-19?

☐*Yes ☐No

If *Yes, the participant may no longer be eligible to participate in the PASS study. Please review the current protocol eligibility criteria.

4. (A) If you are being seen monthly in clinic:
Have you been filling out your symptoms questionnaires at least twice a month and your risk exposure/PPE/social distancing questionnaires once a month?

☐Yes ☐No ☐Not applicable

(B) If you are being seen every three to six months in clinic:
Have you been filling out your risk exposure/PPE/social distancing questionnaires once every three to six months?

☐Yes ☐No ☐Not applicable

CRF ENTRY	CRF QC	DB ENTRY	DB QC
Initial/date	Initial/date	Initial/date	Initial/date

PASS Study Monthly Clinic Visit Questions

SUBJECT ID#: P126-S00-☐☐☐☐ Visit Date: _ _ / _ _ _ / _ _ _ _ Visit #: ____

5. Have you been tested for COVID-19 since your last clinic visit?

☐*Yes ☐No

If *Yes, number of tests done: ☐1 ☐2 ☐3 ☐4 ☐5

COVID-19 TEST #1 (complete for each test)

Date of Test ___ / ___ ___ / ___ ___ ___ *(dd/MMM/yyyy)*

Reason for Testing
☐Symptoms ☐Travel
☐COVID-19 exposure ☐Pre-procedure (medical)
☐Screening test ☐Other________________

Location of Testing
☐WRNMMC COVID-19 outpatient testing facility
☐WRNMMC emergency department
☐Self-collected test
☐Other outpatient facility (name of site) ______________________
☐Other inpatient facility (name of site) ______________________

Type of Test ☐PCR ☐Antigen ☐Do not know

COVID-19 Test Result
☐Negative ☐***Positive**

Did you have any symptoms?
☐***Yes** ☐No

If the COVID-19 test was positive and you had symptoms, complete the next fields.

How many days did your symptoms last (duration of symptoms)?
Please enter a number between **1** and **100** days: ___________

Did you fill out a symptom questionnaire reflecting your most severe symptoms? ☐Yes ☐*No

If *No, please fill out a paper questionnaire in clinic today.

Were you seen in an emergency department for COVID-19? ☐*Yes ☐No

If ***Yes**
Name of site: ________________________

Date of visit: ___ / ___ ___ / ___ ___ ___
(dd/MMM/yyyy)

Were you hospitalized for COVID-19?
☐*Yes ☐No

If ***Yes**
Name of hospital: __________________________

If ***Yes**, what was the hospitalization discharge diagnosis:

____

Date of hospital admission: ___ / ___ ___ / ___ ___ ___
(dd/MMM/yyyy)

Date of hospital discharge: ___ / ___ ___ / ___ ___ ___
(dd/MMM/yyyy)

ENTRIES FOR ADDITIONAL TESTS ARE FOUND AT THE END OF THIS FORM

CRF ENTRY	CRF QC	DB ENTRY	DB QC
Initial/date	Initial/date	Initial/date	Initial/date

PASS Study Monthly Clinic Visit Questions

SUBJECT ID#: P126-S00-☐☐☐☐ Visit Date: _ _ / _ _ _ / _ _ _ _ Visit #: ____

6. Have you started any new medications since your last clinic visit?

☐*Yes ☐No

If *Yes, what are the names and indications of the medications?

Medication Name	Medication Indication
1.	
2.	
3.	
4.	
5.	

7. Have you had any changes to your medical health since your last clinic visit?

☐*Yes ☐No

If *Yes, please describe the changes:

____
____
____
____
____
____

8. Other than you, how many people lived in your household this past month?

Number of people: ________

9. Other than you, how many people in your household have been vaccinated against COVID-19?

Number of people: ________

CRF ENTRY	CRF QC	DB ENTRY	DB QC
Initial/date	Initial/date	Initial/date	Initial/date

PASS Study Monthly Clinic Visit Questions

SUBJECT ID#: P126-S00-☐☐☐☐ Visit Date: _ _ / _ _ _ / _ _ _ _ Visit #: ____

10. Not including you, have any of your household members been diagnosed with COVID-19 since your last clinic visit?

☐*Yes ☐No

If ***Yes**, please tell us the number of individuals who tested positive (other than you):

☐1 ☐2 ☐3 ☐4 ☐5 ☐more than 5

11. Saliva obtained today?

☐Yes ☐*No

If ***No**, please specify reason: ____

Date Obtained: ______________________ *Time Obtained:* ______________________

CRC Initials __________________________

12. Blood obtained today?

☐Yes ☐*No

If ***No**, please specify reason: ____

Number of tubes obtained: ______________

Date Obtained: ______________________ *Time Obtained:* ______________________

CRC Initials __________________________

CRF ENTRY	CRF QC	DB ENTRY	DB QC
Initial/date	Initial/date	Initial/date	Initial/date

PASS Study Monthly Clinic Visit Questions

SUBJECT ID#: P126-S00-☐☐☐☐ Visit Date: _ _ / _ _ _ / _ _ _ _ Visit #: ____

COVID-19 TEST #2 (complete for each test)

Date of Test __ __ / __ __ __ / __ __ __ __ *(dd/MMM/yyyy)*

Reason for Testing
☐ Symptoms ☐ Travel
☐ COVID-19 exposure ☐ Pre-procedure (medical)
☐ Screening test ☐ Other________________

Location of Testing
☐ WRNMMC COVID-19 outpatient testing facility
☐ WRNMMC emergency department
☐ Self-collected test
☐ Other outpatient facility (name of site) ______________________
☐ Other inpatient facility (name of site) ______________________

Type of Test ☐ PCR ☐ Antigen ☐ Do not know

COVID-19 Test Result
☐ Negative ☐ ***Positive**

Did you have any symptoms?
☐ ***Yes** ☐ No

If the COVID-19 test was positive and you had symptoms, complete the next fields.

How many days did your symptoms last (duration of symptoms)?
Please enter a number between **1** and **100** days: ___________

Did you fill out a symptom questionnaire reflecting your most severe symptoms? ☐ Yes ☐ *No

If *No, please fill out a paper questionnaire in clinic today.

Were you seen in an emergency department for COVID-19? ☐ *Yes ☐ No

If ***Yes**
Name of site: ________________________

Date of visit: __ __ / __ __ __ / __ __ __ __
(dd/MMM/yyyy)

Were you hospitalized for COVID-19?
☐ *Yes ☐ No

If ***Yes**
Name of hospital: __________________________

If ***Yes**, what was the hospitalization discharge diagnosis:

____

Date of hospital admission: __ __ / __ __ __ / __ __ __ __
(dd/MMM/yyyy)

Date of hospital discharge: __ __ / __ __ __ / __ __ __ __
(dd/MMM/yyyy)

ENTRIES FOR ADDITIONAL TESTS ARE FOUND AT THE END OF THIS FORM

CRF ENTRY	CRF QC	DB ENTRY	DB QC
Initial/date	Initial/date	Initial/date	Initial/date

PASS Study Monthly Clinic Visit Questions

SUBJECT ID#: P126-S00-☐☐☐☐ Visit Date: _ _ / _ _ _ / _ _ _ _ Visit #: ____

COVID-19 TEST #3 (complete for each test)

Date of Test __ __ / __ __ __ / __ __ __ __ *(dd/MMM/yyyy)*

Reason for Testing
- ☐ Symptoms
- ☐ Travel
- ☐ COVID-19 exposure
- ☐ Pre-procedure (medical)
- ☐ Screening test
- ☐ Other________________

Location of Testing
- ☐ WRNMMC COVID-19 outpatient testing facility
- ☐ WRNMMC emergency department
- ☐ Self-collected test
- ☐ Other outpatient facility (name of site) ______________________
- ☐ Other inpatient facility (name of site) ______________________

Type of Test ☐ PCR ☐ Antigen ☐ Do not know

COVID-19 Test Result
☐ Negative ☐ ***Positive**

Did you have any symptoms?
☐ ***Yes** ☐ No

If the COVID-19 test was positive and you had symptoms, complete the next fields.

How many days did your symptoms last (duration of symptoms)?
Please enter a number between **1** and **100** days: ___________

Did you fill out a symptom questionnaire reflecting your most severe symptoms? ☐ Yes ☐ *No

If *No, please fill out a paper questionnaire in clinic today.

Were you seen in an emergency department for COVID-19? ☐ *Yes ☐ No

If ***Yes**
Name of site: _______________________

Date of visit: __ __ / __ __ __ / __ __ __ __
(dd/MMM/yyyy)

Were you hospitalized for COVID-19?
☐ *Yes ☐ No

If ***Yes**
Name of hospital: _________________________

If ***Yes**, what was the hospitalization discharge diagnosis:

____

Date of hospital admission: __ __ / __ __ __ / __ __ __ __
(dd/MMM/yyyy)

Date of hospital discharge: __ __ / __ __ __ / __ __ __ __
(dd/MMM/yyyy)

ENTRIES FOR ADDITIONAL TESTS ARE FOUND AT THE END OF THIS FORM

CRF ENTRY	CRF QC	DB ENTRY	DB QC
Initial/date	Initial/date	Initial/date	Initial/date

PASS Study Monthly Clinic Visit Questions

SUBJECT ID#: P126-S00-☐☐☐☐ Visit Date: _ _ / _ _ _ / _ _ _ _ Visit #: ____

COVID-19 TEST #4 (complete for each test)

Date of Test __ __ / __ __ __ / __ __ __ __ *(dd/MMM/yyyy)*

Reason for Testing
- ☐Symptoms
- ☐Travel
- ☐COVID-19 exposure
- ☐Pre-procedure (medical)
- ☐Screening test
- ☐Other________________

Location of Testing
- ☐WRNMMC COVID-19 outpatient testing facility
- ☐WRNMMC emergency department
- ☐Self-collected test
- ☐Other outpatient facility (name of site) ______________________
- ☐Other inpatient facility (name of site) _______________________

Type of Test ☐PCR ☐Antigen ☐Do not know

COVID-19 Test Result ☐Negative ☐***Positive**

Did you have any symptoms? ☐***Yes** ☐No

If the COVID-19 test was positive and you had symptoms, complete the next fields.

How many days did your symptoms last (duration of symptoms)?
Please enter a number between **1** and **100** days: ___________

Did you fill out a symptom questionnaire reflecting your most severe symptoms? ☐Yes ☐*No

If *No, please fill out a paper questionnaire in clinic today.

Were you seen in an emergency department for COVID-19? ☐*Yes ☐No

If ***Yes**
Name of site: _______________________

Date of visit: __ __ / __ __ __ / __ __ __ __ *(dd/MMM/yyyy)*

Were you hospitalized for COVID-19? ☐*Yes ☐No

If ***Yes**
Name of hospital: _________________________

If ***Yes**, what was the hospitalization discharge diagnosis:

____

Date of hospital admission: __ __ / __ __ __ / __ __ __ __ *(dd/MMM/yyyy)*

Date of hospital discharge: __ __ / __ __ __ / __ __ __ __ *(dd/MMM/yyyy)*

ENTRIES FOR ADDITIONAL TESTS ARE FOUND AT THE END OF THIS FORM

CRF ENTRY	CRF QC	DB ENTRY	DB QC
Initial/date	Initial/date	Initial/date	Initial/date

PASS Study Monthly Clinic Visit Questions

SUBJECT ID#: P126-S00-☐☐☐☐ Visit Date: _ _ / _ _ _ / _ _ _ _ Visit #: ____

COVID-19 TEST #5 (complete for each test)

Date of Test __ __ / __ __ __ / __ __ __ __ *(dd/MMM/yyyy)*

Reason for Testing
☐Symptoms ☐Travel
☐COVID-19 exposure ☐Pre-procedure (medical)
☐Screening test ☐Other________________

Location of Testing
☐WRNMMC COVID-19 outpatient testing facility
☐WRNMMC emergency department
☐Self-collected test
☐Other outpatient facility (name of site) ______________________
☐Other inpatient facility (name of site) ______________________

Type of Test ☐PCR ☐Antigen ☐Do not know

COVID-19 Test Result
☐Negative ☐***Positive**

Did you have any symptoms?
☐***Yes** ☐No

If the COVID-19 test was positive and you had symptoms, complete the next fields.

How many days did your symptoms last (duration of symptoms)?
Please enter a number between **1** and **100** days: ___________

Did you fill out a symptom questionnaire reflecting your most severe symptoms? ☐Yes ☐*No

If *No, please fill out a paper questionnaire in clinic today.

Were you seen in an emergency department for COVID-19? ☐*Yes ☐No

If ***Yes**
Name of site: ________________________

Date of visit: __ __ / __ __ __ / __ __ __ __
(dd/MMM/yyyy)

Were you hospitalized for COVID-19?
☐*Yes ☐No

If ***Yes**
Name of hospital: __________________________

If ***Yes**, what was the hospitalization discharge diagnosis:

____

Date of hospital admission: __ __ / __ __ __ / __ __ __ __
(dd/MMM/yyyy)

Date of hospital discharge: __ __ / __ __ __ / __ __ __ __
(dd/MMM/yyyy)

ENTRIES FOR ADDITIONAL TESTS ARE FOUND AT THE END OF THIS FORM

CRF ENTRY	CRF QC	DB ENTRY	DB QC
Initial/date	Initial/date	Initial/date	Initial/date
