# Supplementary material for: Prospective Assessment of SARS-CoV-2 Seroconversion (PASS) study: an observational cohort study of SARS-CoV-2 infection and vaccination in healthcare workers
Source: BMC Infect Dis. 2021 Jun 9;21:544. doi: 10.1186/s12879-021-06233-1 (PMC8188741; doi:10.1186/s12879-021-06233-1)
Supplement: Supplementary file 6 — Additional file 6. Email reminder - symptoms questionnaire. [file 12879_2021_6233_MOESM6_ESM.pdf]

Dear PASS Study Participant,

The attached email link will take you to the SYMPTOMS questionnaire.

1) Please fill out this questionnaire at least once every two weeks.

2) Also, please fill out this questionnaire any and every day you have any symptoms asked about on this questionnaire if the symptoms differ from your usual baseline health.

3) Finally, please remember to report to the WRNMMC COVID-19 Testing Center to be tested for COVID-19 every time you develop any of these symptoms.

   Symptoms listed on the questionnaire:
   - *runny or drippy nose, congested or stuffy nose, sinus pressure,*
   - *scratchy or itchy throat, sore or painful throat, difficulty swallowing,*
   - *teary or watery eyes, sore or painful eyes, eyes sensitive to light,*
   - *trouble breathing, chest congestion, chest tightness, cough,*
   - *nausea, stomach ache,*
   - *dizziness,*
   - *head congestion, headache,*
   - *lack of appetite,*
   - *sleeping more than usual,*
   - *body aches or pains, feeling weak or tired,*
   - *chills or shivering, feeling hot, feeling cold, sweating,*
   - *loss of taste, and loss of smell.*

4) If you have a positive COVID-19 test notify the [redacted] as soon as possible at:

[redacted]

Sincerely,

The PASS Study Team
