# Supplementary material for: Prospective Assessment of SARS-CoV-2 Seroconversion (PASS) study: an observational cohort study of SARS-CoV-2 infection and vaccination in healthcare workers
Source: BMC Infect Dis. 2021 Jun 9;21:544. doi: 10.1186/s12879-021-06233-1 (PMC8188741; doi:10.1186/s12879-021-06233-1)
Supplement: Supplementary file 7 — Additional file 7. Risk exposure questionnaire. [file 12879_2021_6233_MOESM7_ESM.pdf]

PASS Study – Risk Exposure, PPE Use, and Social Distancing

SUBJECT ID#: P126-S00-☐☐☐☐ Visit Date: _ _ / _ _ _ / _ _ _ _ Visit #: ____

Risk Exposure, PPE Use, and Social Distancing (to be completed at enrollment visit and monthly)

Risk Exposure Questions at Work

1. Since the last questionnaire, how many days were you working at the hospital? [value: 0-15], if=0, no more work questions

2. Since the last questionnaire, have you:
   - ☐ had direct interaction with COVID-19 positive patients
   - ☐ had direct interaction with COVID-19 presumptive patients (no test results)

3. Since the last questionnaire, have you conducted high risk activities on COVID-19 patients, such as intubation, airway suctioning, administration of nebulizer therapy, or advanced cardiac life support (responding to a code)? Yes/No

Risk Exposure Questions at Home

1. Since the last questionnaire, has any member of your household tested positive for COVID-19? Yes/No

2. Since the last questionnaire, has any member of your household had symptoms consistent with COVID-19 but not been tested? Yes/No

PPE and social distancing at work

1. Since the last questionnaire, how consistent are you with using recommended PPE during patient interactions?
   (none of the time, less than half of the time, about half of the time, more than half of the time, all of the time)

2. Since the last questionnaire, (in the hospital or in the clinics), how often are you wearing a face mask when you are not directly interacting with patients?
   (none of the time, less than half of the time, about half of the time, more than half of the time, all of the time)

3. How consistently are you applying hand sanitizer and/or washing hands before and after interacting with patients at the hospital?
   (none of the time, less than half of the time, about half of the time, more than half of the time, all of the time)

4. List any other precautionary social distancing or disinfecting measures you are taking at work that are not discussed here.

PASS Study – Risk Exposure, PPE Use, and Social Distancing

SUBJECT ID#: P126-S00-☐☐☐☐Visit Date: _ _ / _ _ _ / _ _ _ _ Visit #: ____

PPE and Social Distancing outside work:

5. Since the last questionnaire, how often did you wear a mask while conducting activities in businesses outside of your home (ex. a grocery store) and in crowded locations (ex. subway)? (none of the time, less than half of the time, about half of the time, more than half of the time, all of the time)

6. How often did you wear a mask while participating in solitary outdoor activities such as walking, jogging, in non-crowded locations? (none of the time, less than half of the time, about half of the time, more than half of the time, all of the time)

7. What type of mask(s) do you typically use outside of work for these activities?
   - ☐ Homemade/non-medical grade cloth mask
   - ☐ Sterilizable surgical mask
   - ☐ disposable surgical mask
   - ☐ N95 respirator

8. In the past month, how often have you been:	0 times	1-2 times	3-5 times	6-10 times	> 10 times
In a grocery store	☐	☐	☐	☐	☐
In a restaurant	☐	☐	☐	☐	☐
In a gym or workout studio	☐	☐	☐	☐	☐
On a bus, train, or subway	☐	☐	☐	☐	☐
At a sporting event	☐	☐	☐	☐	☐
At a music, dance, or theater production	☐	☐	☐	☐	☐

9. In the past month, how often have you been to gatherings with people outside your immediate household at one time (select all that apply)?

Size of gathering (not including yourself)	0 times	1-2 times	3-5 times	6-10 times	> 10 times
1-2 people	☐	☐	☐	☐	☐
3-5 people	☐	☐	☐	☐	☐
6-10 people	☐	☐	☐	☐	☐
11-50 people	☐	☐	☐	☐	☐
>50 people	☐	☐	☐	☐	☐

10. Are you consistently practicing social distancing while outside of your home (not at work)? Yes/No

11. Are you routinely disinfecting mail/packages that are delivered to your home? Yes/No/Sometimes

12. List any other precautionary social distancing or disinfecting measures you are taking at home that are not answered here.
