# Supplementary material for: Prospective Assessment of SARS-CoV-2 Seroconversion (PASS) study: an observational cohort study of SARS-CoV-2 infection and vaccination in healthcare workers
Source: BMC Infect Dis. 2021 Jun 9;21:544. doi: 10.1186/s12879-021-06233-1 (PMC8188741; doi:10.1186/s12879-021-06233-1)
Supplement: Supplementary file 8 — Additional file 8. Email reminder - risk exposure questionnaire. [file 12879_2021_6233_MOESM8_ESM.pdf]

Dear PASS Study Participant,

The attached email link will take you to the RISK EXPOSURE/PPE USE/SOCIAL DISTANCING questionnaire.

Please fill out this questionnaire at least once every month. The questionnaire will inquire about your COVID-19 risk exposures, PPE use, and social distancing practices at work and in settings outside work.

Please answer the questions as honestly and accurately as possible. While data obtained from the entire study cohort of up to 300 healthcare workers may be shared with WRNMMC leadership, be assured that individual responses will not be.

Additionally, please remember to come to your monthly PASS Study clinic visit. If you do not remember the date and time of your next PASS Study clinic visit, please contact the [redacted] at:

[redacted]

Sincerely,

The PASS Study Team
