# Supplementary material for: Prospective Assessment of SARS-CoV-2 Seroconversion (PASS) study: an observational cohort study of SARS-CoV-2 infection and vaccination in healthcare workers
Source: BMC Infect Dis. 2021 Jun 9;21:544. doi: 10.1186/s12879-021-06233-1 (PMC8188741; doi:10.1186/s12879-021-06233-1)
Supplement: Supplementary file 9 — Additional file 9. COVID-19 vaccination history form. [file 12879_2021_6233_MOESM9_ESM.pdf]

COVID-19 VACCINATION HISTORY FORM

SUBJECT ID#: P126-S00- ☐☐☐☐ Visit Date: _ _ / _ _ _ / _ _ _ _ Visit #: ____

This form will be filled out either while at the CTC clinic or will be sent to the study subjects electronically at clinic visits when a subject reports having received a COVID-19 vaccine (after both first dose and booster).

1. *What date did you receive your COVID-19 vaccine?*

   Date: __ __ / __ __ __ / __ __ __ __ (dd/MMM/yyyy)

2. *Was this the first or second vaccination with this vaccine?*

   ☐ *1st*

   ☐ *2nd*

3. *What company manufactured the vaccine?*

   ☐ *Pfizer/BioNTech*

   ☐ *Moderna*

   ☐ *Oxford/AztraZeneca*

   ☐ *Novavax*

   ☐ *Johnson and Johnson*

   ☐ *do not know*

   ☐ *other ____________________*

4. *Where did you receive your vaccine?*

   ☐ *WRNMMC*

   ☐ *other facility __________________*

CRF ENTRY	CRF QC	DB ENTRY	DB QC
Initial/date	Initial/date	Initial/date	Initial/date

COVID-19 VACCINATION HISTORY FORM

SUBJECT ID#: P126-S00- ☐☐☐☐ Visit Date: _ _ / _ _ _ / _ _ _ _ Visit #: ____

5. *Please rate the extent to which you had any of the following symptoms after vaccination.*

	**Not at all**	**A little bit**	**Somewhat**	**Quite a bit**	**Very much**
POINTS	0	1	2	3	4
Pain at injection site	☐	☐	☐	☐	☐
Soreness at injection site	☐	☐	☐	☐	☐
Redness at injection site	☐	☐	☐	☐	☐
Weak or tired	☐	☐	☐	☐	☐
Headache	☐	☐	☐	☐	☐
Body aches or pains	☐	☐	☐	☐	☐
Felt hot	☐	☐	☐	☐	☐
Felt cold	☐	☐	☐	☐	☐
Chills or shivering	☐	☐	☐	☐	☐
Joint pains	☐	☐	☐	☐	☐
Felt nauseous (feeling like you want to throw up)	☐	☐	☐	☐	☐
Swollen lymph nodes	☐	☐	☐	☐	☐

CRF ENTRY	CRF QC	DB ENTRY	DB QC
Initial/date	Initial/date	Initial/date	Initial/date

COVID-19 VACCINATION HISTORY FORM

SUBJECT ID#: P126-S00- ☐☐☐☐ Visit Date: _ _ / _ _ _ / _ _ _ _ Visit #: ____

6. For how many days did you have symptoms?

☐0 (no symptoms)

☐1 ☐2 ☐3 ☐4 ☐5 ☐6 ☐7 ☐8 ☐9 ☐10 ☐more than 10 days

7. Did you track these symptoms on the PASS study symptoms questionnaire?

☐Yes ☐No

CRF ENTRY	CRF QC	DB ENTRY	DB QC
Initial/date	Initial/date	Initial/date	Initial/date
